# CP-EXCEL:  A feasibility randomised controlled trial of an online exercise programme for adults with cerebral palsy

**DOI:** 10.12688/hrbopenres.14150.1

**Published:** 2025-07-31

**Authors:** Manjula Manikandan, Pauline Hunter-Graham, Ailish McGahey, Miriam Creeger, Rebecca Walters, Aisling Walsh, Karen McConnell, Claire Kerr, Claire Kenny, Éabha Wall, Fiona Weldon, Jessica Burke, Kevin Bravender, Rachel Byrne, Jennifer Ryan

**Affiliations:** 1 CP-Life Research Centre, School of Physiotherapy, Royal College of Surgeons in Ireland, Dublin, Dublin, Dublin 2, Ireland; 2 Mae Murray Foundation, Northern Ireland, UK; 3 Central Remedial Clinic, Dublin, Ireland; 4 UP- The Adult Cerebral Palsy movement, London, UK; 5 University College London Hospitals NHS Foundation Trust, London, England, UK; 6 Department of Public Health and Epidemiology, Royal College of Surgeons in Ireland, Dublin, Ireland; 7 School of Nursing and Midwifery, Queen's University Belfast, Belfast, UK; 8 Public and Patient Involvement, contributor, Ireland; 9 Strategies for change Co-ordinator, Independent Living Movement Ireland, Dublin, Ireland; 10 The cerebral palsy foundation, New York, USA

**Keywords:** Cerebral palsy, Adults, online exercise programme, fitness

## Abstract

**Introduction:**

Adults with cerebral palsy (CP) are at increased risk of non-communicable diseases and often experience secondary complications as they age. Physical activity can mitigate these risks and improve well-being. However, adults with CP face significant challenges in accessing structured, tailored exercise programmes due to limited service availability and environmental barriers. A group online exercise programme may overcome some of the barriers to accessing exercise faced by adults with CP. CP-EXCEL aims to evaluate the feasibility of an online exercise programme for adults with CP. The study will examine the demand, implementation, practicality, adaptability, acceptability, and potential efficacy of delivering the online exercise programme to adults with CP in Ireland.

**Methods:**

This feasibility randomised controlled trial will recruit 60 adults with CP. Participants will be randomly assigned to either an eight-week online exercise intervention or a control group. The feasibility of the programme will be evaluated across five key domains: 1) demand as indicated by recruitment rate; 2) implementation as indicated by attendance; 3) practicality as indicated by number and type of adverse events ; 4) adaptability, by examining how the programme accommodates varying levels of mobility and associated impairments; and 5) acceptability explored through semi-structured interviews with participants. To assess preliminary efficacy, the Patient-Reported Outcomes Measurement Information System (PROMIS) will be used to measure changes in physical, mental, and social functioning at baseline and post-intervention. Quantitative outcomes will be analysed using linear mixed models, and qualitative data will be analysed using framework analysis.

**Conclusion:**

This study will provide valuable insights into the feasibility of an online exercise programme tailored for adults with CP.

## Background

Cerebral palsy (CP) is a lifelong neurological condition characterised by impaired movement, often in combination with cognitive, speech and sensory impairments ^1^. In comparison to those without CP, adults with CP are less physically active and at higher risk of developing non-communicable diseases ^2, 3^. Further, many adults with CP experience pain, fatigue, decline in walking ability, reduced muscle flexibility and strength, and increased risk of falls with age ^4– 6^. These complications may restrict their capacity to engage in daily activities and lead to a diminished sense of participation ^7^.

Participation in exercise may reduce the risk of developing non-communicable disease and manage complications associated with CP and ageing ^7, 8^. However, adults with CP face challenges trying to access exercise programmes in the community, including lack of appropriate gym equipment, lack of transport, and inaccessible facilities ^9^. Further, people with CP want support from physiotherapists with knowledge of CP who can adapt the exercises to suit their ability and provide advice on how to manage the complications of CP ^9, 10^. However, a recent study highlighted that 23% of adults with CP in Ireland have an unmet need for physiotherapy ^11^. The most common reasons adults with CP accessed physiotherapy were for mobility decline, stiffness and pain ^12^.

The increased use of online healthcare delivery since Covid-19 ^13^ provides an opportunity to address some of the barriers that individuals with CP face in accessing physiotherapy-led exercise programmes. Online programmes may also have benefits over in-person programmes for people with CP such as increased opportunity for peer support and access to specialist knowledge regardless of geography, and enhanced comfort in their own home ^14^. 

Recognising these challenges and opportunities, the CP-EXCEL study aims to evaluate the feasibility of an online exercise programme for adults with CP. If results indicate the programme is feasible, it may provide a sustainable approach to addressing the unmet need for physiotherapy among adults with CP and improve their health and participation.

### Aim

In this project, we will evaluate the feasibility of an online exercise programme for adults with CP. Six domains of an evidence-based framework for feasibility studies will be evaluated: demand, implementation, practicality, potential efficacy testing, adaptability and acceptability.

### Objectives are to

- Describe recruitment rate.
- Describe fidelity to the programme.
- Assess number and type of adverse events.
- Explore acceptability and adaptability of the online exercise programme to adults with CP.
- Investigate effects of the programme on physical, mental and social outcomes.

## Methods

### Study design

This is a feasibility randomised controlled trial. This protocol is registered on clinicaltrials.gov (NCT06983782) available at [ https://clinicaltrials.gov/study/NCT06983782], registered 20 ^th^ May 2025, last updated on 20 ^th^ June 2025. The protocol is guided by the Standard Protocol Items: Recommendations for Interventional Trials (SPIRIT) guidelines (Appendix 1) Extended data ^15^.

### Participants

Participants will be recruited through newsletters and social media of organisations that offer services and support to adults with CP in the community across the island of Ireland, including the Central Remedial Clinic (CRC), Irish Wheelchair Association (IWA) and Mae Murray Foundation.

Sixty adults with CP will be recruited in total, with thirty participants allocated to the intervention group and thirty to the control group. We will include adults aged 18 and over with CP residing on the island of Ireland in any Gross Motor Function Classification System (GMFCS) level ^16^. Individuals with severe intellectual disability will be excluded where sufficient adaptations cannot be made to support them to access the online platform and follow study instructions. We will also exclude Adults with unstable medical conditions (e.g. heart conditions).

### Recruitment

**Figure 1.**
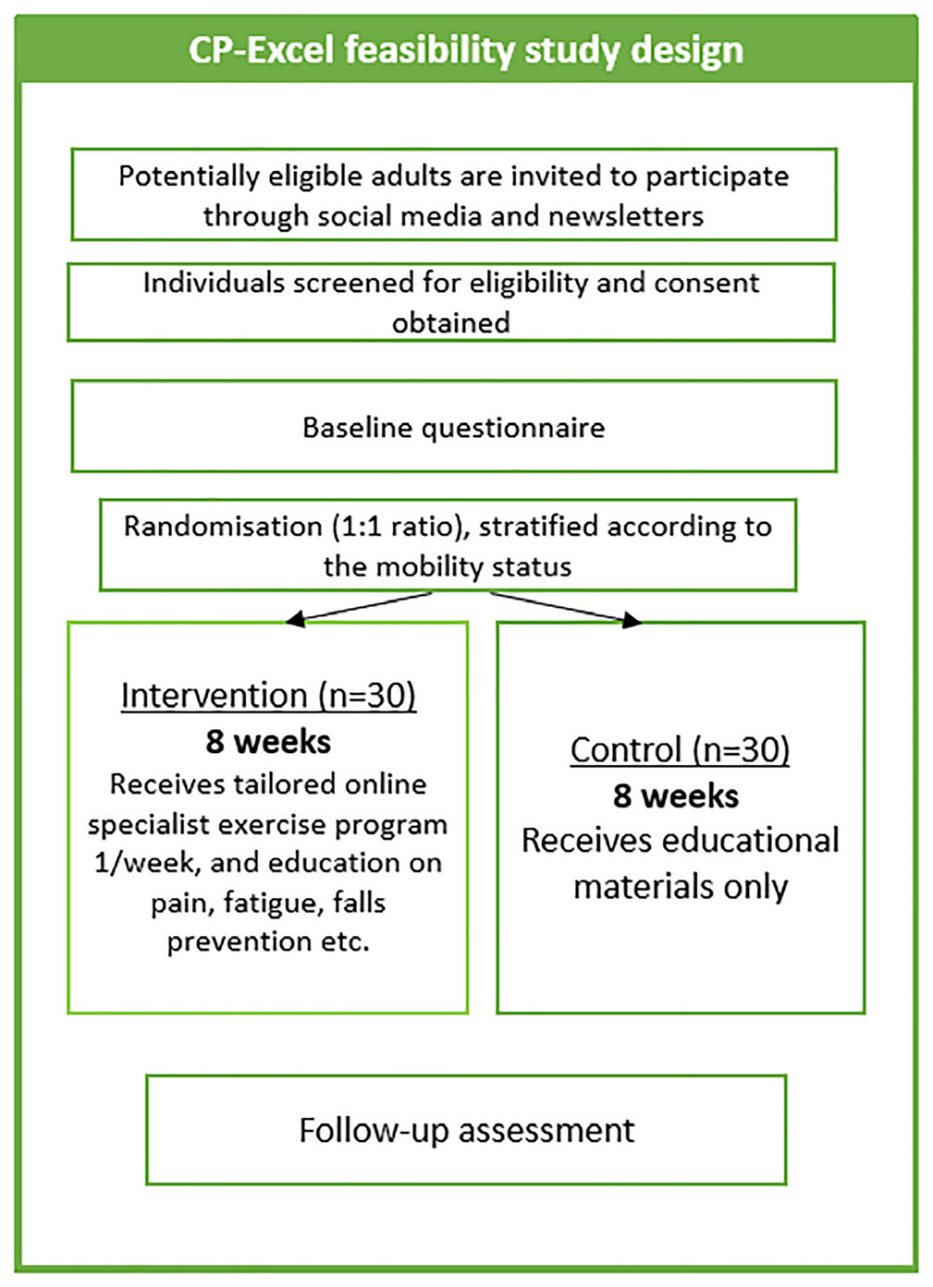
CP-Excel study design.

The study procedure is outlined in Figure 1. If interested in taking part, adults can contact the post-doctoral research fellow who will introduce the study, provide a participant information leaflet (PIL), and answer any questions. The PIL includes detailed information about the study, eligibility criteria, the consent and withdrawal processes, confidentiality, data protection, data storage, and the researchers’ contact details.

All participants will complete a brief screening survey which includes personal details (name, email/phone number), and any health concerns that may prevent them from safely engaging in the exercise programme. After the screening process, participants who meet the eligibility criteria will be asked to provide informed written or electronic consent to the post-doctoral research fellow.

Following screening and consent, all participants will undergo a baseline assessment. Participants will be randomised to the intervention or control group, and then re-assessed at the end of week 8.

### Randomisation

Following the baseline assessment, participants will be randomly allocated to either an intervention or control group in a 1:1 ratio. Stratification will be based on their mobility status: GMFCS Level I-III (with or without walking aids) and GMFCS Levels IV-V (who use wheelchairs). The random allocation will be generated by a computer in random permuted blocks of 2 or 4, managed by an individual independent to the study. The allocation sequence will be stored in sequentially numbered, opaque, sealed envelopes: a researcher will draw an envelope in sequence and inform the participant of their group assignment.

### Risk assessment

Participants in the intervention group will be contacted to schedule a one-to-one video call for a comprehensive risk assessment. The risk assessment was developed from resources shared by experts in developing risk assessments for online exercise classes in Ireland. This was then reviewed by a group of physiotherapists and a personal trainer with expertise in CP. The risk assessment includes:

- Next of Kin - name and telephone number, in case we need to contact someone in an emergency
- Physical activity Readiness questionnaire (PAR-Q+) to assess the readiness to participate in the exercise programme ^17^.
- Mobility and falls history
- Balance assessment will be conducted using the 4-stage balance test (for GMFCS Levels I-III, as shown in Appendix 2-extended data), which evaluates static balance ^18^. Additionally, the sit-to-stand test (5 times) will be used to assess functional balance ^19^.
- Environmental assessment for safe space. This assessment will evaluate the participant’s environment for potential trip hazards, the availability of a safe exercise space, proper screen setup, adequate lighting, and the presence of support persons if needed. Additional considerations will include ensuring access to a supportive chair or other necessary equipment, and technical support for accessing the online platform if required. Participants will also be required to sign a safety checklist following the assessment.

## Intervention group: Online exercise programme

 The intervention is an 8-week online exercise and education programme consisting of an online exercise class once per week, in accordance with exercise prescription guidelines for people with CP ^7^. The programme will consist of eight group sessions, each lasting 60 minutes. Each session will include (i) 5–8 minutes for warm-up, a 30 minute exercise programme, 5 minutes for cool-down, and (ii) 15–20 minutes of interactive learning focused on a topic of relevance to adults with CP.

The online exercise class will focus on mobility, strength and balance exercises for major muscle groups, based on the most common reasons that adults with CP seek physiotherapy ^12^. Participants will complete up to 10–11 exercises in each class. The exercises may be completed in sitting or standing depending on the person’s ability. Participants will complete between 2 and 4 sets of 6–15 repetitions of each exercise. Examples of exercises include overhead reach, Table or box push-ups, Lateral arm raises, Knee up, hip back or wheelchair dip, side step or hip out, sit to stand, squat, knee straight/bend, heel raises and seated rowing.

The 15–20 minute interactive learning component of each weekly online class will consist of educational materials on exercise, nutrition, mobility changes, fatigue, optimising bone health, pain management, mental health, sleep and physical activity maintenance (one topic per week).

The programme will be delivered by a physiotherapist (post-doctoral research fellow) and a specialist personal trainer in exercise for long-term neurological conditions. The programme content was developed with input from physiotherapists and personal trainers with expertise in CP, and from the Public and Patient Involvement (PPI) contributors who are adults with CP. The exercise programme will be piloted with PPI contributors in advance of the intervention commencing: any necessary refinements will be made based on feedback received.

### Control group

Participants allocated to the control group will receive the educational materials on exercise, nutrition, mobility changes, fatigue, optimising bone health, pain management, mental health, sleep and physical activity maintenance at the start of the study.

## Data collection

### Outcomes

Six domains of an evidence-based framework for feasibility studies will be evaluated: demand, implementation, practicality, potential efficacy testing, adaptability and acceptability ^20^ ( Table 1).

**Table 1.** CP-Excel outcomes.

Domain	CP-Excel Outcomes
Demand	Recruitment rate
Implementation	Fidelity to the programme: Attendance at each class and completion of the exercise programme.
Practicality	Recording of adverse events.
Adaptability	Semi-structured interviews with adults with CP
Acceptability	Semi-structured interviews with adults with CP
Potential efficacy	Physical, mental and social outcomes

### Assessments

All participants will complete self-reported assessments at baseline (0 weeks) and immediately after participation in the eight week programme. Assessments may be completed online, on the phone, in person with the post-doctoral research fellow or on paper. Participants who complete paper versions will be given a freepost envelope to return the completed forms. The online outcome measures will be hosted on REDCap (Research Electronic Data Capture), an online survey platform.

The following data will be collected at baseline only:

1. Socio-demographic
  a. Age
  b. Gender

2. Condition-specific information
  a. Type of CP
  b. GMFCS level
  c. Intellectual disability
  d. Secondary impairments

The data shown in Table 3 will be collected at baseline and follow-up:

### Demand

Demand will be assessed by recording recruitment rate to the programme. This will include recording the total number of people who responded to invitations or advertisements to participate in the study, the number of potentially eligible participants identified over a 3-month period, the proportion of eligible participants who provided consent to participate, and the reasons for exclusions or decisions to decline participation.

### Implementation

Implementation will be assessed by recording:

- Attendance at sessions to evaluate fidelity to intervention receipt.
- Completion of exercise programme to evaluate fidelity of intervention content by asking participants in the intervention group at each week and control group at the follow-up assessment.

### Practicality

Practicality will be assessed by recording adverse events by asking participants in both groups at each assessments.

**An Adverse Event (AE)** is defined as any untoward medical occurrence affecting a participant that does not necessarily have a causal relationship with the intervention ( https://www.ct-toolkit.ac.uk/glossary).

**A serious adverse event (SAE)** is defined untoward medical occurrence/effect that:

(a) results in death;

(b) is life-threatening;

(c) requires hospitalisation or prolongation of existing hospitalisation;

(d) results in persistent or significant disability or incapacity;

**Expected adverse events**

Due to the benign nature of the physical activity intervention, no serious adverse events are anticipated. However, the following non-serious expected adverse events, typically associated with increased physical activity, may occur and will be recorded in the Case Report Form:

- Delayed Onset Muscle Soreness (DOMS)
- Mild fatigue

An **Unexpected Adverse Event** is defined as an event whose nature or severity is not consistent with expected outcomes of the intervention and is not listed in the protocol as an expected AE.

Participants in the intervention group will be instructed to contact the research team if they experience any AEs during the study. In addition, at each exercise session, participants will be asked whether they have experienced any AEs since their last contact.

All reported adverse events will be documented. The seriousness, expectedness, and causality of each event will be assessed by the research team.

### Acceptability

*Acceptability* will be assessed by conducting semi-structured interviews with 15 adults with CP who participated in the online exercise programme using the theoretical framework of acceptability ^21^ to inform topic guides and data analysis. The seven component constructs that represent acceptability are detailed in Table 2 and include: affective attitude, burden, perceived effectiveness, ethicality, intervention coherence, opportunity costs, and self-efficacy ^22^.

**Table 2.** Theoretical framework of acceptability components ^22^.

Components	Description
Affective Attitude	How an individual feels about the intervention
Burden	The perceived amount of effort that is required to participate in the intervention
Ethicality	The extent to which the intervention has good fit with an individual’s value system.
Intervention Coherence	The extent to which the participant understands the intervention and how it works
Opportunity Costs	The extent to which benefits, profits or values must be given up to engage in the intervention.
Perceived Effectiveness	The extent to which the intervention is perceived as likely to achieve its purpose.
Self-Efficacy	The participant’s confidence that they can perform the behavior required to participate in the intervention.

Topic guides will be developed in collaboration with clinical expert team and PPI contributors including adults with CP. The topic guides will be piloted with at least two adults with CP. Adaptations will be made to ensure the interviews are accessible and inclusive. This may include providing the topic guide in advance, using alternative systems of communication (using images as prompts), and pictorial memory aids. We will also offer conducting the interviews over two or more segments to minimise fatigue. The interviews will be conducted in person, by telephone or by Microsoft Teams, based on participant preference. In person interviews will be conducted in a private, accessible and comfortable setting that is free from distractions and allows for privacy and confidentiality. Interviews will be audio-recorded and transcribed verbatim.

### Adaptability

Adaptability will be assessed by exploring the adaptability of the programme for associated impairments and mobility levels during the semi-structured interview.

### Efficacy

Efficacy will be assessed by investigating changes in outcomes from baseline to 8-weeks ( Table 3). The Patient-Reported Outcomes Measurement Information System (PROMIS) will be used to measure changes in physical, mental, and social functioning at baseline and post-intervention ^23– 26^.

**Table 3.** Outcomes.

Outcome	Outcome measure
Mobility	Functional mobility scale ^28^
Physical function	PROMIS- Physical function- Short form 10a
Pain	Self-reported questionnaire
Pain intensity	PROMIS- Pain intensity form
Pain quality	PROMIS Neuropathic pain quality 5a PROMIS Nociceptive Pain quality 5a
Pain interference	PROMIS- Pain interference – Short Form-8a
Fatigue	PROMIS- Fatigue- Short form 13a (FACIT fatigue)
Sleep	PROMIS- Sleep disturbance- Short Form 4a
Self-efficacy for managing daily activities	PROMIS- Self-efficacy for managing chronic conditions- managing daily activities- Short Form 8a
Self-efficacy for managing emotions	PROMIS- Self-efficacy for managing chronic conditions- managing emotions- Short Form 8a
Depression	PROMIS- Emotional Distress – Depression- Short Form 8a
Participation in social roles and activities	PROMIS- Ability to participate in social roles and activities- Short Form 8a

## Data analysis

### Quantitative data analysis

To determine the demand of the programme, descriptive statistics will be used to report the number and proportion of participants who responded to invitations, potentially eligible participants identified over a 3-month period who meet the inclusion criteria, who agree to participate in the study, excluded from the study and who drop-out during the study.

To determine the fidelity of the programme, descriptive statistics will be used to report the number of sessions attended by participants and those who completed the exercise contents.

To determine the efficacy, we will explore changes in outcomes over time using Generalised Estimating Equations with appropriate link functions depending on the outcome. Where a participant is missing data, they will be excluded from the analysis. Multiple imputation methods will not be applied.

### Qualitative data analysis

Data from semi-structured interviews will be analysed through Framework analysis. Framework analysis is appropriate for this study as we have pre-defined components of acceptability (Sekhon *et al.*, 2017) that we wish to explore but are also open to emergence of additional themes. Adaptability of the programme for associated impairments and mobility level will also be explored from this interview data.

Framework analysis involves seven iterative stages: Transcription, familiarisation, coding, developing a working analytical framework, applying the analytical framework, charting, and interpretation ^27^. The post-doctoral research fellow will read a sample of transcripts until familiarity with the data is established. Then will independently develop provisional codes. The codes will be reviewed, discussed and refined in collaboration with the project management group (PMG), who will also read selected transcripts to support the development of a working analytical framework. Once the framework is established, the post-doctoral research fellow will apply it to all transcripts. Any issues arising during this process will be discussed with the PMG, before finalising the framework. Data will then be arranged into charts that summarise themes, issues and individual responses. Finally, the post-doctoral research fellow will present the emerging categories and themes to the advisory group and PPI for discussion. We will use strategies to enhance trustworthiness of the findings, such as negative case analysis, peer-debriefing and reflexivity.

### Withdrawals

Participants who withdraw before the intervention will continue to receive standard usual care. Participants will be encouraged to allow data that have been collected before withdrawal to be used in the analyses. However, if consent to use data is also withdrawn, then these will be discarded. Participant withdrawals will be recorded in a Change of Status form.

## Discussion

This protocol presents the CP-EXCEL feasibility trial, designed to test an online exercise programme for adults with CP. Adults with CP often experience chronic pain, fatigue, and reduced mobility, worsened by limited access to appropriate rehabilitation age ^4– 6, 12^. Barriers such as inaccessible environments, lack of transport, and few CP-trained professionals hinder regular physical activity ^12, 29^. The CP-EXCEL programme aims to address these issues through structured, home-based online delivery that includes exercise and education, potentially offering a more accessible and scalable solution.

This study will provide important insights into the feasibility of such an intervention, including recruitment, retention, adherence, and outcome measure acceptability. Co-development with adults with CP and healthcare professionals enhances its relevance and user-centred design. A key limitation, however, is the small sample size, which is standard for feasibility studies. As a result, the trial is not powered to detect statistically significant effects, but outcome data will inform the design and sample size requirements of a future, larger trial.

## Conclusion

The CP-EXCEL trial will provide essential guidance for developing and evaluating an accessible, online exercise programme for adults with CP. These findings will support the design of a full-scale trial to assess long-term outcomes and clinical effectiveness.

## Ethics

The study will be conducted in full conformance with the principles of the Declaration of Helsinki. Ethics approval was obtained from the Royal College of Surgeons in Ireland Research Ethics Committee (REC202501020- 13 ^th^ May 2025, Amendments: REC202505023- 27 ^th^ May 2025) and awaiting CRC REC approval. All researchers working on the study will receive training in Good Clinical Practice guidelines. Explicit, informed, written consent will be obtained from adults with CP.

## Patient and Public Involvement (PPI) group

The PPI group is composed of five adults with CP living in Ireland who are co-authors on this paper.

## Dissemination

It is our intention to disseminate the results of the study as widely as possible. This will be through at least one publication in a peer-reviewed journal, and through presentations at National and International conferences. Publications will follow the CONSORT guidelines. Authorship will follow international guidelines. A dissemination plan will be developed in the early phases of the study in collaboration with the PMG, CEG and PPI groups.

## Data management

A detailed data management plan outlining data entry, storage and preservation is currently under review.

## Protocol version control

**Table:** 

**Version**	**Date**	**Description**
**V1.0**	**13/05/2025**	**Approved by ethics committee**
**V1.1**	**27/05/2025**	**Amendments approved by ethics committee**
		

## Data Availability

No data are associated with this article. The following extended data are available via Figshare: https://doi.org/10.6084/m9.figshare.29328932 ^30^ Appendix 1: SPIRIT Checklist Appendix 2: Four-Stage Balance Test Consent Form Participant Information Leaflet (example) SPIRIT checklist for CP-EXCEL: A feasibility randomised controlled trial of an online exercise programme for adults with cerebral palsy (Appendix 1 https://doi.org/10.6084/m9.figshare.29328932).
