# CP-EXCEL:  A feasibility randomised controlled trial of an online exercise programme for adults with cerebral palsy

**DOI:** 10.12688/hrbopenres.14150.2

**Published:** 2026-05-05

**Authors:** Manjula Manikandan, Pauline Hunter-Graham, Ailish McGahey, Miriam Creeger, Rebecca Walters, Aisling Walsh, Karen McConnell, Claire Kerr, Claire Kenny, Éabha Wall, Fiona Weldon, Jessica Burke, Kevin Bravender, Rachel Byrne, Jennifer Ryan

**Affiliations:** 1 CP-Life Research Centre, School of Physiotherapy, Royal College of Surgeons in Ireland, Dublin, Dublin, Dublin 2, Ireland; 2 Mae Murray Foundation, Northern Ireland, UK; 3 Central Remedial Clinic, Dublin, Ireland; 4 UP- The Adult Cerebral Palsy movement, London, UK; 5 University College London Hospitals NHS Foundation Trust, London, England, UK; 6 Department of Public Health and Epidemiology, Royal College of Surgeons in Ireland, Dublin, Ireland; 7 School of Nursing and Midwifery, Queen's University Belfast, Belfast, UK; 8 Public and Patient Involvement, contributor, Ireland; 9 Strategies for change Co-ordinator, Independent Living Movement Ireland, Dublin, Ireland; 10 The cerebral palsy foundation, New York, USA

**Keywords:** Cerebral palsy, Adults, online exercise programme, fitness

## Abstract

**Introduction:**

Adults with cerebral palsy (CP) are at increased risk of non-communicable diseases and often experience secondary complications as they age. Physical activity can mitigate these risks and improve well-being. However, adults with CP face significant challenges in accessing structured, tailored exercise programmes due to limited service availability and environmental barriers. A group online exercise programme may overcome some of the barriers to accessing exercise faced by adults with CP. CP-EXCEL aims to evaluate the feasibility of an online exercise programme for adults with CP. The study will examine the demand, implementation, practicality, adaptability, acceptability, and potential efficacy of delivering the online exercise programme to adults with CP in Ireland.

**Methods:**

This feasibility randomised controlled trial will recruit 60 adults with CP. Participants will be randomly assigned to either an eight-week online exercise intervention or a control group. The feasibility of the programme will be evaluated across five key domains: 1) demand as indicated by recruitment rate; 2) implementation as indicated by attendance; 3) practicality as indicated by number and type of adverse events; 4) adaptability, by examining how the programme accommodates varying levels of mobility and associated impairments; and 5) acceptability explored through semi-structured interviews with participants. To assess preliminary efficacy, the Patient-Reported Outcomes Measurement Information System (PROMIS) will be used to measure changes in physical, mental, and social functioning at baseline and post-intervention. Quantitative outcomes will be analysed using linear mixed models, and qualitative data will be analysed using framework analysis.

**Conclusion:**

This study will provide valuable insights into the feasibility of an online exercise programme tailored for adults with CP And inform the design of a future fully powered randomised controlled trial.

## Background

Cerebral palsy (CP) is a lifelong neurological condition characterised by impaired movement, often in combination with cognitive, speech and sensory impairments. ^1^ In comparison to those without CP, adults with CP are less physically active and at higher risk of developing non-communicable diseases. ^2^ ^,^ ^3^ Further, many adults with CP experience pain, fatigue, decline in walking ability, reduced muscle flexibility and strength, and increased risk of falls with age. ^4^ ^–^ ^6^ These complications may restrict their capacity to engage in daily activities and lead to a diminished sense of participation. ^7^

Participation in exercise may reduce the risk of developing non-communicable disease and manage complications associated with CP and ageing. ^7^ ^,^ ^8^ Existing evidence suggests that individuals with CP can safely participate in structured exercise programmes incorporating aerobic and resistance training, typically performed 2–4 times per week. ^7^ Resistance training commonly involves 2–4 sets of 6–15 repetitions, with intensity and progression tailored to individual ability. ^7^ Emerging evidence also suggests that higher-intensity or power-based training may provide additional benefits for muscle strength and functional mobility in children and youth with CP. ^9^ ^–^ ^11^ The suitability of these approaches may vary depending on factors such as age, mobility level, safety considerations, and the ability to deliver such training within group-based or remote settings. However, adults with CP face challenges trying to access exercise programmes in the community, including lack of appropriate gym equipment, lack of transport, and inaccessible facilities. ^12^ Further, people with CP want support from physiotherapists with knowledge of CP who can adapt the exercises to suit their ability and provide advice on how to manage the complications of CP. ^12^ ^,^ ^13^ However, a recent study highlighted that 23% of adults with CP in Ireland have an unmet need for physiotherapy. ^14^ The most common reasons adults with CP accessed physiotherapy were for mobility decline, stiffness and pain. ^15^

In addition to physiotherapy-led interventions, evidence suggests that adults with CP have limited knowledge and support to effectively manage their condition and associated secondary complications. ^16^ Many report seeking information from the internet or peers rather than from health professionals. ^16^ Educational approaches can provide information on secondary complications associated with ageing in CP, which may support individuals to better understand and manage these challenges. Such approaches may complement exercise-based interventions by promoting self-management and supporting ongoing engagement in physical activity.

The increased use of online healthcare delivery since Covid-19 ^17^ provides an opportunity to address some of the barriers that individuals with CP face in accessing physiotherapy-led exercise programmes. Online programmes may also have benefits over in-person programmes for people with CP such as increased opportunity for peer support and access to specialist knowledge regardless of geography, and enhanced comfort in their own home. ^18^ These group-based formats may also support social participation and mental wellbeing, which are closely linked to quality of life outcomes. ^19^ ^,^ ^20^

Recognising these challenges and opportunities, the CP-EXCEL study aims to evaluate the feasibility of an online exercise programme for adults with CP. CP-EXCEL refers to the feasibility randomised controlled trial designed to assess the delivery, acceptability and preliminary outcomes of this intervention. Findings from this study will inform the design and conduct of a future fully powered randomised controlled trial. If results indicate the programme is feasible, it may provide a sustainable approach to addressing the unmet need for physiotherapy among adults with CP and improve their health and participation.

### Aim

In this project, we will evaluate the feasibility of an online group-based exercise and education programme for adults with CP, compared to an education component. Six domains of an evidence-based framework for feasibility studies will be evaluated: demand, implementation, practicality, potential efficacy testing, adaptability and acceptability.

### Objectives are to

• Describe recruitment rate.
• Describe fidelity to the programme.
• Assess number and type of adverse events.
• Explore acceptability and adaptability of the online exercise programme to adults with CP.
• Investigate effects of the programme on physical, mental and social outcomes to inform the design of a future fully powered randomised controlled trial.

## Methods

### Study design

This is a feasibility randomised controlled trial. Designed to assess the feasibility of study procedures, including recruitment, randomisation, retention, intervention delivery, and outcome assessment, rather than to evaluate intervention effectiveness. Participants will be randomised to either an 8-week online exercise programme or a control group. Outcome measures will be collected at baseline and immediately post-intervention. The study is not powered to detect statistically significant differences in outcomes, and any analysis of outcomes will be exploratory in nature. This protocol is registered on clinicaltrials.gov ( NCT06983782) available at [ https://clinicaltrials.gov/study/NCT06983782], registered 20 ^th^ May 2025, last updated on 20 ^th^ June 2025. The protocol is guided by the Standard Protocol Items: Recommendations for Interventional Trials (SPIRIT) guidelines (Appendix 1) Extended data. ^22^

### Participants

Participants will be recruited through newsletters and social media of organisations that offer services and support to adults with CP in the community across the island of Ireland, including the Central Remedial Clinic (CRC), Irish Wheelchair Association (IWA) and Mae Murray Foundation.

Sixty adults with CP will be recruited in total, with thirty participants allocated to the intervention group and thirty to the control group. This sample size is appropriate for a feasibility randomised controlled trial and is not based on a formal power calculation. It is informed by the study aim to assess feasibility parameters and by estimated recruitment rates of 5–10% from previous studies involving adults with CP in Ireland. ^14^

We will include adults aged 18 and over with CP residing on the island of Ireland in any Gross Motor Function Classification System (GMFCS) level. ^23^ Individuals with severe intellectual disability will be excluded where sufficient adaptations cannot be made to support them to access the online platform and follow study instructions. We will also exclude Adults with unstable medical conditions (e.g. heart conditions).

Participants will be supported to engage in the study using accessible materials and flexible communication approaches (e.g., simplified information or verbal explanation), and may be assisted by a family member or support person where appropriate (e.g., to support consent or participation in online sessions).

### Recruitment

**Figure 1.**
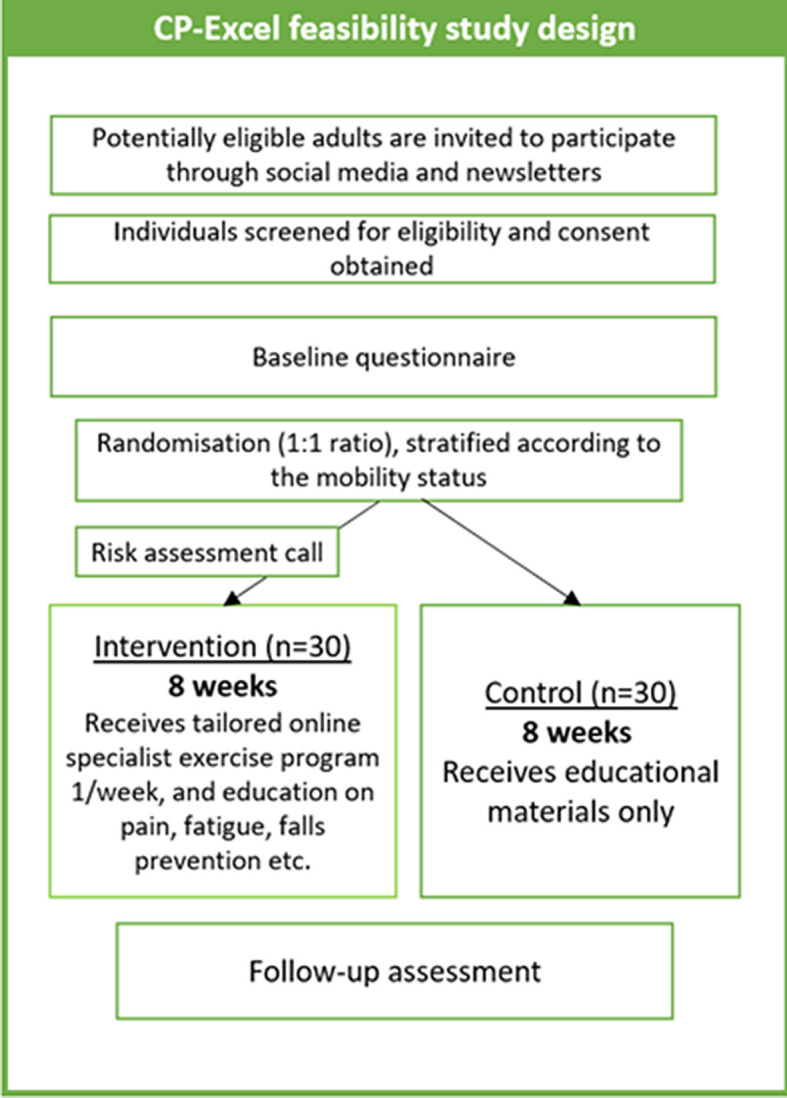
CP-Excel study design.

The study procedure is outlined in Figure 1. If interested in taking part, adults can contact the post-doctoral research fellow who will introduce the study, provide a participant information leaflet (PIL), and answer any questions. The PIL includes detailed information about the study, eligibility criteria, the consent and withdrawal processes, confidentiality, data protection, data storage, and the researchers’ contact details.

All participants will complete a brief screening survey which includes personal details (name, email/phone number), and any health concerns that may prevent them from safely engaging in the exercise programme. Medical stability will be assessed through self-report in this survey, including information on current health conditions and any contraindications to exercise, and clarified through follow-up discussion with the research team where required. The ability to participate in the study will also be assessed during screening and initial interaction with the researcher, considering communication, cognitive ability, and ability to follow instructions. Where appropriate, the potential for support from a family member or caregiver and the feasibility of making adaptations will be considered to facilitate participation. After the screening process, participants who meet the eligibility criteria will be asked to provide informed written or electronic consent to the post-doctoral research fellow.

Following screening and consent, all participants will undergo a baseline assessment via an online survey. Participants will be randomised to the intervention or control group, and then re-assessed at the end of week 8.

### Randomisation

Following the baseline assessment, participants will be randomly allocated to either an intervention or control group in a 1:1 ratio. Stratification will be based on their mobility status: GMFCS Level I-III (with or without walking aids) and GMFCS Levels IV-V (who use wheelchairs). The random allocation will be generated by a computer in random permuted blocks of 2 or 4, and implemented automatically within REDCap (Research Electronic Data Capture). Allocation concealment will be ensured as the sequence is embedded within REDCap and not accessible to the research team prior to assignment.

### Risk assessment

Participants in the intervention group will be contacted to schedule a one-to-one video call for a comprehensive virtual risk assessment prior to commencing the intervention. The risk assessment was developed from resources shared by experts in developing risk assessments for online exercise classes in Ireland. This was then reviewed by a group of physiotherapists and a personal trainer with expertise in CP. The risk assessment includes:

• Next of Kin - name and telephone number, in case we need to contact someone in an emergency.
• Physical activity Readiness questionnaire (PAR-Q+) to assess the readiness to participate in the exercise programme. ^24^ Responses to the PAR-Q+ and other screening components will be reviewed by the research team, and any identified concerns will be discussed with the participant to determine whether safe participation can be supported.
• Mobility and falls history.
• Balance assessment will be conducted using the 4-stage balance test (for GMFCS Levels I-III, as shown in Appendix 2-extended data), which evaluates static balance. ^25^ The 4-stage balance test has demonstrated reliability and has been used in remote assessment contexts. ^26^ ^,^ ^27^ While these measures have demonstrated reliability and have been used in remote contexts, there is limited evidence for their formal validation in adults with CP when administered virtually. Although not formally validated for online use in CP, standardised instructions and adaptations will be applied to ensure consistent and safe assessment across GMFCS levels. These assessments will be conducted via video call, with guidance provided on camera positioning to ensure clear observation of movement. Participants will be instructed to perform assessments near a stable support (e.g., chair or wall) to enhance safety. Additionally, the sit-to-stand test (5 times) will be used to assess functional balance. ^28^ Findings from the balance and functional assessments will be used to inform the individualisation of the exercise programme, including appropriate exercise selection, level of difficulty, and required adaptations, to ensure safe and appropriate participation.
• Environmental assessment for safe space. This assessment will evaluate the participant’s environment for potential trip hazards, the availability of a safe exercise space, proper screen setup, adequate lighting, and the presence of support persons if needed. Additional considerations will include ensuring access to a supportive chair or other necessary equipment, and technical support for accessing the online platform if required. Participants will also be required to sign a safety checklist following the assessment.
• Where risks are identified, efforts will be made to adapt the environment and exercises to support safe participation. Participants will not be excluded solely based on environmental constraints where reasonable adaptations can be made; however, if safety cannot be ensured, participants will not proceed to the intervention component of the study.

## Intervention group: Online exercise and education programme

The intervention is an 8-week online exercise and education programme consisting of an online exercise class once per week, in accordance with exercise prescription guidelines for people with CP. ^7^ The programme will consist of eight weekly group sessions, each lasting 60 minutes. Each session will include (i) 5–8 minutes for warm-up, a 30 minute exercise programme, 5 minutes for cool-down, and (ii) 15–20 minutes of interactive learning focused on a topic of relevance to adults with CP.

The online exercise class will focus on mobility, strength and balance exercises for major muscle groups, based on the most common reasons that adults with CP seek physiotherapy. ^15^ Balance will be both explicitly targeted and integrated within functional exercises throughout the programme. Participants will complete up to 10–11 exercises in each class. In standing, balance will be challenged through functional tasks such as sit-to-stand, squats, side stepping, toe taps and exercises involving weight shifting or changes in base of support. The exercises may be completed in sitting or standing depending on the person’s ability. For participants exercising in sitting, balance will be addressed through trunk control and postural stability activities, such as unsupported sitting, reaching tasks, and dynamic upper limb movements performed within a safe and supported environment. The intervention will be tailored to individual ability, with exercises adapted based on mobility level (GMFCS I-V), upper limb function, associated impairments (e.g. communication, vision, or hearing) and risk assessment findings, while maintaining a consistent overall structure. Where required, participants may be supported by a family member or caregiver to assist with communication, technology use, or safe participation. Participants will complete between 2 and 4 sets of 6–15 repetitions of each exercise. Exercises will focus on functional, task-oriented movements targeting mobility, strength, and balance. The programme will emphasise controlled, submaximal effort and movement quality rather than maximal or high-velocity (power-based) training. Exercise intensity will be individualised and guided by participant ability, with participants encouraged to work at a moderate level that is challenging but safe.

Participants will also be encouraged to repeat the exercises independently twice per week between sessions. Participants will be provided with access to private exercise videos demonstrating the exercises performed during the online sessions to support independent completion. Adherence to the intervention will be recorded through attendance at online sessions and a brief self-reported survey capturing whether participants completed the prescribed exercises independently at least twice per week. The number of sets and repetitions performed during supervised sessions will also be recorded, with progression guided by participant ability over the 8-week programme. Progression will be achieved by modifying repetitions, sets, range of movement, level of support, and exercise complexity, rather than through one-repetition maximum testing. Examples of exercises include overhead reach, Table or box push-ups, Lateral arm raises, Knee up, hip back or wheelchair dip, side step or hip out, sit to stand, squat, knee straight/bend, heel raises and seated rowing as well as balance-focused activities such as weight-shifting and reaching tasks. For example, exercises such as squats may be performed in standing with or without support or adapted to seated alternatives (e.g., sit-to-stand or wheelchair-based upper body movements) to accommodate different mobility levels. Exercises will be demonstrated with options and progressions to enable participation within a group setting while accommodating individual differences. Verbal cueing and demonstration will be used to guide correct technique, pacing, and safe execution of movements during each session.

The 15–20 minute interactive learning component of each weekly online class will consist of educational materials on exercise, nutrition, mobility changes, fatigue, optimising bone health, pain management, mental health, sleep and physical activity maintenance (one topic per week). Educational materials and exercises will be delivered during the online class. The educational content is consistent across both study groups; however, in the intervention group it is delivered interactively during weekly sessions.

The programme will be delivered by a physiotherapist (post-doctoral research fellow) and a specialist personal trainer in exercise for long-term neurological conditions. The programme content was developed with input from physiotherapists and personal trainers with expertise in CP, and from the Public and Patient Involvement (PPI) contributors who are adults with CP. The exercise programme will be piloted with PPI contributors in advance of the intervention commencing: any necessary refinements will be made based on feedback received. This will include refinement of adaptations to support delivery across a range of abilities and needs. During the intervention, adaptations will be informed by participant feedback and session experience. All adaptations made during programme delivery (e.g., modifications to exercises, level of support, or delivery approach) will be documented prospectively by the researcher delivering the intervention. The researcher delivering the programme will review sessions, document adaptations and implement any necessary adaptations in subsequent group classes. These documented adaptations will inform ongoing refinement of the intervention, interpretation of feasibility outcomes, and future implementation.

Detailed intervention materials, including exercise and educational content, will be made available alongside the publication of the study findings, informed by the feasibility outcomes.

### Control group

Participants allocated to the control group will receive the same educational materials as those in the intervention group. The educational content is consistent across both groups; however, in the intervention group it is delivered interactively within supervised sessions, while in the control group it is provided in a self-directed format. These will cover topics including exercise, nutrition, mobility changes, fatigue, optimising bone health, pain management, mental health, sleep and physical activity maintenance. These materials will be delivered weekly via email over the 8 week study period. Participants in the control group will not be prescribed a structured exercise programme during the study period. Following completion of the 8-week programme, participants in the control group will receive access to the same exercise videos and will be offered the opportunity to take part in an optional online exercise class.

Participants in both groups will be asked to continue their usual activities and will not be instructed to modify or stop any existing physical activity. The intervention is described in accordance with the TIDieR (Template for Intervention Description and Replication) checklist to support replicability. ^29^

## Data collection

### Outcomes

Six domains of an evidence-based framework for feasibility studies will be evaluated: demand, implementation, practicality, potential efficacy testing, adaptability and acceptability ^21^ ( Table 1). Each domain will be assessed using predefined metrics as outlined in Table 1, including recruitment rate (demand), attendance and exercise completion (implementation), adverse events (practicality), qualitative interviews exploring acceptability and adaptability, and changes in physical, mental and social outcomes (potential efficacy). These findings will inform progression to a larger, fully powered trial.

**Table 1.** CP-Excel outcomes.

Domain	CP-Excel Outcomes
Demand	Recruitment rate
Implementation	Fidelity to the programme: Attendance at each class and completion of the exercise programme
Practicality	Recording of adverse events
Adaptability	Semi-structured interviews with adults with CP
Acceptability	Semi-structured interviews with adults with CP
Potential efficacy	Physical, mental and social outcomes

### Assessments

All participants will complete self-reported assessments at baseline (0 weeks) and immediately after participation in the eight week programme. Assessments may be completed online, on the phone, in person with the post-doctoral research fellow or on paper. Participants who complete paper versions will be given a freepost envelope to return the completed forms. The online outcome measures will be hosted on REDCap, an online survey platform.

The following data will be collected at baseline only:

1. Socio-demographic

a. Age
b. Gender

2. Condition-specific information

a. Type of CP
b. GMFCS level
c. Intellectual disability
d. Secondary impairments

The data shown in Table 3 will be collected at baseline and follow-up:

### Demand

Demand will be assessed by recording recruitment rate to the programme. This will include recording the total number of people who responded to invitations or advertisements to participate in the study, the number of potentially eligible participants identified over a 3-month period, the proportion of eligible participants who provided consent to participate, and the reasons for exclusions or decisions to decline participation.

### Implementation

Implementation will be assessed by recording:

• Attendance at sessions to evaluate fidelity to intervention receipt.
• Self-reported completion of the prescribed exercise programme to evaluate fidelity of intervention content. Participants in the intervention group will complete a brief weekly survey indicating whether they completed the prescribed exercises independently at least twice per week in addition to the supervised session.

Practicality will be assessed by recording adverse events reported by participants in both groups at each assessment time point.

**An Adverse Event (AE)** is defined as any untoward medical occurrence affecting a participant that does not necessarily have a causal relationship with the intervention ( https://www.ct-toolkit.ac.uk/glossary).

**A serious adverse event (SAE)** is defined untoward medical occurrence/effect that:

(a) results in death;
(b) is life-threatening;
(c) requires hospitalisation or prolongation of existing hospitalisation;
(d) results in persistent or significant disability or incapacity.

### Expected adverse events

Due to the benign nature of the physical activity intervention, no serious adverse events are anticipated. However, the following non-serious expected adverse events, typically associated with increased physical activity, may occur and will be recorded in the Case Report Form:

• Delayed Onset Muscle Soreness (DOMS)
• Mild fatigue

An **Unexpected Adverse Event** is defined as an event whose nature or severity is not consistent with expected outcomes of the intervention and is not listed in the protocol as an expected AE.

Participants in the intervention group will be instructed to contact the research team if they experience any AEs during the study. In addition, at each exercise session, participants will be asked whether they have experienced any AEs since their last contact.

All reported adverse events will be documented. The seriousness, expectedness, and causality of each event will be assessed by the research team.

### Acceptability

*Acceptability* will be assessed by conducting semi-structured interviews with 15 adults with CP who participated in the online exercise programme using the theoretical framework of acceptability ^30^ to inform topic guides and data analysis. The seven component constructs that represent acceptability are detailed in Table 2 and include: affective attitude, burden, perceived effectiveness, ethicality, intervention coherence, opportunity costs, and self-efficacy. ^31^

**Table 2.** Theoretical framework of acceptability components. ^31^

Components	Description
Affective Attitude	How an individual feels about the intervention
Burden	The perceived amount of effort that is required to participate in the intervention
Ethicality	The extent to which the intervention has good fit with an individual’s value system.
Intervention Coherence	The extent to which the participant understands the intervention and how it works
Opportunity Costs	The extent to which benefits, profits or values must be given up to engage in the intervention.
Perceived Effectiveness	The extent to which the intervention is perceived as likely to achieve its purpose.
Self-Efficacy	The participant’s confidence that they can perform the behavior required to participate in the intervention.

Topic guides will be developed in collaboration with clinical expert team and PPI contributors including adults with CP. The topic guides will be piloted with at least two adults with CP. Adaptations will be made to ensure the interviews are accessible and inclusive. This may include providing the topic guide in advance, using alternative systems of communication (using images as prompts), and pictorial memory aids. We will also offer conducting the interviews over two or more segments to minimise fatigue. The interviews will be conducted in person, by telephone or by Microsoft Teams, based on participant preference. In person interviews will be conducted in a private, accessible and comfortable setting that is free from distractions and allows for privacy and confidentiality. Interviews will be audio-recorded and transcribed verbatim.

### Adaptability

Adaptability will be assessed by exploring the adaptability of the programme for associated impairments and mobility levels during the semi-structured interview.

### Efficacy

Efficacy will be assessed by investigating changes in outcomes from baseline to 8-weeks ( Table 3). The Patient-Reported Outcomes Measurement Information System (PROMIS) will be used to measure changes in physical, mental, and social functioning at baseline and post-intervention. ^32^ ^–^ ^35^ PROMIS measures are standardised self-reported instruments assessing domains such as physical function, pain, fatigue, sleep, mental health, and participation, typically reported as T-scores, with the direction of scoring dependent on the domain. The Functional Mobility Scale (FMS) assesses mobility across different distances and use of assistive devices, ^36^ and will be collected at both baseline and 8-week follow-up to assess changes over time. These measures were selected due to their relevance to the intervention, feasibility for remote administration, and suitability for adults with CP. Both have established psychometric properties, including reliability and validity, and have been used in neurological populations. ^32^ ^–^ ^36^ Further details on scoring and interpretation are provided in Table 3.

**Table 3.** Outcomes.

Outcome	Outcome measure
Mobility	Functional mobility scale ^36^
Physical function	PROMIS-Physical function-Short form 10a
Pain	Self-reported questionnaire
Pain intensity	PROMIS-Pain intensity form
Pain quality	PROMIS Neuropathic pain quality 5a PROMIS Nociceptive Pain quality 5a
Pain interference	PROMIS-Pain interference – Short Form-8a
Fatigue	PROMIS-Fatigue-Short form 13a (FACIT fatigue)
Sleep	PROMIS-Sleep disturbance-Short Form 4a
Self-efficacy for managing daily activities	PROMIS-Self-efficacy for managing chronic conditions-managing daily activities-Short Form 8a
Self-efficacy for managing emotions	PROMIS-Self-efficacy for managing chronic conditions-managing emotions-Short Form 8a
Depression	PROMIS-Emotional Distress – Depression-Short Form 8a
Participation in social roles and activities	PROMIS-Ability to participate in social roles and activities-Short Form 8a

## Data analysis

### Quantitative data analysis

To determine the demand of the programme, descriptive statistics will be used to report the number and proportion of participants who responded to invitations, potentially eligible participants identified over a 3-month period who meet the inclusion criteria, who agree to participate in the study, excluded from the study and who drop-out during the study.

To determine the fidelity of the programme, descriptive statistics will be used to report the number of sessions attended by participants and those who completed the exercise contents.

As this is a feasibility study, analyses of outcomes will be exploratory and are not intended to provide definitive evidence of effectiveness. To determine potential efficacy, we will explore changes in outcomes over time. Continuous PROMIS outcomes will be analysed using linear mixed models to examine changes between groups over time. The Functional Mobility Scale (FMS), an ordinal measure, will be analysed descriptively and explored using appropriate non-parametric or ordinal methods where feasible. Outcomes will be interpreted descriptively in line with the feasibility design, with reference to established scoring approaches for PROMIS measures where available. Where data are missing, analysis will be conducted on available cases, and patterns of missingness will be explored descriptively. Multiple imputation methods will not be applied. STATA version 18 will be used for analysis.

### Qualitative data analysis

Data from semi-structured interviews will be analysed through Framework analysis. Framework analysis is appropriate for this study as we have pre-defined components of acceptability (Sekhon *et al.*, 2017) that we wish to explore but are also open to emergence of additional themes. Adaptability of the programme for associated impairments and mobility level will also be explored from this interview data. Participant experiences of both the exercise and health education components of the intervention, and their interaction, will also be explored.

Framework analysis involves seven iterative stages: Transcription, familiarisation, coding, developing a working analytical framework, applying the analytical framework, charting, and interpretation. ^37^ The post-doctoral research fellow will read a sample of transcripts until familiarity with the data is established. Then will independently develop provisional codes. The codes will be reviewed, discussed and refined in collaboration with the project management group (PMG), who will also read selected transcripts to support the development of a working analytical framework. Once the framework is established, the post-doctoral research fellow will apply it to all transcripts. Any issues arising during this process will be discussed with the PMG, before finalising the framework. Data will then be arranged into charts that summarise themes, issues and individual responses. Finally, the post-doctoral research fellow will present the emerging categories and themes to the advisory group and PPI for discussion. We will use strategies to enhance trustworthiness of the findings, such as negative case analysis, peer-debriefing and reflexivity. NVivo 12 Pro (Lumivero, Denver, CO, USA) will be used to support data management and analysis.

### Withdrawals

Participants who withdraw prior to commencing the intervention will continue to receive standard usual care, defined as any healthcare, physiotherapy, exercise, or community-based services they would typically access independently as part of their routine care. Participants will not be instructed to modify or discontinue any existing care or physical activity during the study. Participants will be encouraged to allow data that have been collected before withdrawal to be used in the analyses. However, if consent to use data is also withdrawn, then these will be discarded. Participant withdrawals will be recorded in a Change of Status form. Participants who withdraw will not be replaced.

## Discussion

This protocol presents the CP-EXCEL feasibility trial, designed to test an online exercise programme for adults with CP. Adults with CP often experience chronic pain, fatigue, and reduced mobility, worsened by limited access to appropriate rehabilitation age. ^4^ ^–^ ^6^ ^,^ ^15^ Barriers such as inaccessible environments, lack of transport, and few CP-trained professionals hinder regular physical activity. ^15^ ^,^ ^38^ The CP-EXCEL programme aims to address these issues through structured, home-based online delivery that includes exercise and education, potentially offering a more accessible and scalable solution.

This study will provide important insights into the feasibility of such an intervention, including recruitment, retention, adherence, and outcome measure acceptability. Co-development with adults with CP and healthcare professionals enhances its relevance and user-centred design. A key limitation, however, is the small sample size, which is standard for feasibility studies. As a result, the trial is not powered to detect statistically significant effects, but outcome data will inform the design and sample size requirements of a future, larger trial.

## Conclusion

The CP-EXCEL trial will provide essential guidance for developing and evaluating an accessible, online exercise programme for adults with CP. These findings will support the design of a full-scale trial to assess long-term outcomes and clinical effectiveness.

## Ethics

The study will be conducted in full conformance with the principles of the Declaration of Helsinki. Ethics approval was obtained from the Royal College of Surgeons in Ireland Research Ethics Committee (REC202501020–13 ^th^ May 2025, Amendments: REC202505023–27 ^th^ May 2025) and awaiting CRC REC approval. All researchers working on the study will receive training in Good Clinical Practice guidelines. Explicit, informed, written consent will be obtained from adults with CP.

## Patient and Public Involvement (PPI) group

The PPI group is composed of five adults with CP living in Ireland who are co-authors on this paper. PPI contributors have been involved in the development of the research question, study design, study materials (including the information leaflet, consent forms, survey and interview topic guide), as well as piloting of the intervention and survey measures. They will continue to be involved in advising on recruitment strategies, accessibility, and delivery of the intervention content, as well as interpreting findings and supporting dissemination. Their input will contribute to refining the intervention design, improving accessibility, and enhancing the overall acceptability and feasibility of the study procedures. PPI involvement will be reported in line with the GRIPP2-SF reporting guidelines in the final study publication.

## Dissemination

It is our intention to disseminate the results of the study as widely as possible. This will be through at least one publication in a peer-reviewed journal, and through presentations at National and International conferences, and sharing outputs with adults with CP, families, and health professionals and wider stakeholders (e.g., fitness professionals and policy makers) via executive and lay summaries, webinars, and online platforms. Publications will follow the CONSORT guidelines. Authorship will follow international guidelines. A dissemination plan will be co-developed in the early phases of the study in collaboration with the PMG, CEG and PPI groups to ensure outputs are accessible. Relevant and tailored to different audiences. Intervention materials will also be shared to support replication and implementation in future research and practice. Findings from the study, including qualitative data, will be used to identify potential barriers and facilitators to implementation, which will inform strategies to support uptake of the programme in clinical and community settings.

A summary of the study findings will be provided to all participants in an accessible format. This summary will be co-developed with adults with CP on the research team to ensure it is clear, relevant, and highlights how participant involvement has contributed to understanding and improving practice. This activity complements other dissemination strategies, including executive and lay summaries, webinars, and online materials.

## Data management

A detailed data management plan outlining data entry, storage and preservation is currently under review.

## Protocol version control

**Table:** 

Version	Date	Description
**V1.0**	**13/05/2025**	**Approved by ethics committee**
**V1.1**	**27/05/2025**	**Amendments approved by ethics committee**

## Data availability

No data are associated with this article.

### Extended data

The following extended data are available via Figshare: https://doi.org/10.6084/m9.figshare.29328932 ^39^

Appendix 1: SPIRIT Checklist.

Appendix 2: Four-Stage Balance Test

• Consent Form
• Participant Information Leaflet (example)

## Reporting guidelines

SPIRIT checklist for CP-EXCEL: A feasibility randomised controlled trial of an online exercise programme for adults with cerebral palsy (Appendix 1 https://doi.org/10.6084/m9.figshare.29328932).
