# Supplementary material for: CP-EXCEL:  A feasibility randomised controlled trial of an online exercise programme for adults with cerebral palsy
Source: HRB Open Res. 2026 Jun 23;8:85. Originally published 2025 Jul 31. [Version 3] doi: 10.12688/hrbopenres.14150.3 (PMC13221627; doi:10.12688/hrbopenres.14150.3)
Supplement: Peer review history

# Reviewer report: Reviewer response for version 2

**DOI:** 10.21956/hrbopenres.15921.r55128

**Published:** 2026-05-29

**Authors:** Danielle Bellows

**Affiliations:** 1 School of Physical Therapy, Massachusetts College of Pharmacy and Health Sciences, Worcester, USA

I appreciate the opportunity to review the revised study protocol for the CP-EXCEL feasibility randomized controlled trial. The authors have responded thoughtfully and comprehensively to the feedback provided in the initial review. The clarity of the methods,  particularly around the dual-purpose framing of the feasibility aims, intervention description, risk assessment procedures, outcome measure justification, and the knowledge translation and dissemination plan, substantially strengthens the transparency and replicability of the protocol. The expanded description of patient and public involvement in dissemination and knowledge translation, including the planned co-development with the PPI of a participant-facing summary of findings, is a particular strength and reflects genuine engagement with, and relevance to, the individuals this research is designed to serve.

 Two minor improvements to this study protocol are recommended.

 First, the text references Table 3 for further details on outcome measure scoring and interpretation. However, the table as presented does not appear to contain this information. Please ensure Table 3 is updated or revise the in-text reference accordingly.

 Second, there are a few instances in the manuscript where text appears to have been split into two incomplete sentences rather than retained as a single coherent sentence. (e.g., “This is a feasibility randomised controlled trial. Designed to assess the feasibility of study procedures…”, and  “A dissemination plan will be co-developed in the early phases of the study in collaboration with the PMG, CEG and PPI groups to ensure outputs are accessible. Relevant and tailored to different audiences.”)   A careful proofread specifically for sentence-level continuity is recommended.

 I commend the research team on the quality and rigor of this work and wish them well in the successful implementation of the study. I look forward to reading the study findings and learning how this important work ultimately informs the design of the fully powered trial and the broader field of physical activity intervention for adults with cerebral palsy.

Is the study design appropriate for the research question?

Yes

Is the rationale for, and objectives of, the study clearly described?

Yes

Are sufficient details of the methods provided to allow replication by others?

Partly

Are the datasets clearly presented in a useable and accessible format?

Not applicable

Reviewer Expertise:

Implementation Science Capacity Building, Implementation Practice, Knowledge Brokering, Adults with Childhood Onset- Disability, Inclusive Fitness, Qualitative, Mixed Methods, and Participatory Research in Pediatric Rehabilitation.

I confirm that I have read this submission and believe that I have an appropriate level of expertise to confirm that it is of an acceptable scientific standard.

# Response

**Published:** 2026-06-15

**Authors:** Manjula Manikandan

**Affiliations:** Royal College of Surgeons in Ireland, Ireland

Thank you for highlighting this inconsistency. We have removed the statement referring readers to Table 3 for further details on scoring and interpretation, as this information was not included in the table.

 Thank you for identifying these issues. We have carefully proofread the manuscript and revised the affected sections to improve sentence structure, continuity, and readability throughout the manuscript.

# Reviewer report: Reviewer response for version 2

**DOI:** 10.21956/hrbopenres.15921.r55129

**Published:** 2026-05-22

**Authors:** Margaret Wallen

**Affiliations:** 1 Australian Catholic University, Sydney, Australia

Thank you for the opportunity to complete a second review of this study, CP-EXCEL, which explores an online physical activity programme for adults with cerebral palsy. My feedback following the initial review consisted of suggestions to enhance reporting of various aspects of the study to support replicability and critical review. The quality of the authors’ work is reflected in their subsequent attention to these suggestions and reporting of the planned study. I have three very minor comments.

 Table 3 doesn’t contain the detail promised by: “Further details on scoring and interpretation are provided in Table 3.”

 The evidence-based feasibility framework should be referenced in the AIM of the study.

 Clarify what the safety checklist is that participants sign, and its purpose, and how it relates to the “Environmental assessment for safe space”

 The reporting of the methods for this study clearly indicate the rigour around the work. I wish the researchers well is securing CRC REC approval and successful study implementation. I look forward to reading the final report and subsequent implementation of the findings.

Is the study design appropriate for the research question?

Yes

Is the rationale for, and objectives of, the study clearly described?

Yes

Are sufficient details of the methods provided to allow replication by others?

Partly

Are the datasets clearly presented in a useable and accessible format?

Not applicable

Reviewer Expertise:

NA

I confirm that I have read this submission and believe that I have an appropriate level of expertise to confirm that it is of an acceptable scientific standard.

# Response

**Published:** 2026-06-15

**Authors:** Manjula Manikandan

**Affiliations:** Royal College of Surgeons in Ireland, Ireland

Thank you for highlighting this inconsistency. We have removed the statement “Further details on scoring and interpretation are provided in Table 3” as these details were not included in Table 3.

 Thank you for this suggestion. We have revised the Aim section to explicitly reference the evidence-based feasibility framework used to guide the study.

 We have clarified the purpose of the safety checklist and its relationship to the environmental assessment. The checklist is reviewed and signed following the assessment to confirm that participants have received and understood the safety recommendations discussed regarding the exercise environment, equipment, support persons if required, exercise warning signs, and exercising within their individual ability level.

# Reviewer report: Reviewer response for version 1

**DOI:** 10.21956/hrbopenres.15562.r51560

**Published:** 2025-12-31

**Authors:** Danielle Bellows

**Affiliations:** 1 School of Physical Therapy, Massachusetts College of Pharmacy and Health Sciences, Worcester, USA

**SUMMARY**

 This randomized controlled trial will assess the feasibility of an online group exercise program for adults with cerebral palsy. Results will inform a larger, fully powered study.

 60 adults (18+ years) with cerebral palsy living in Ireland, GMFCS levels I-V, will be recruited. Following baseline screening for complex medical or intellectual conditions, participants will be randomized via concealed allocation (1:1, random permuted blocks) to either an 8-week online exercise program with a weekly education component or to a comparison group receiving only education at the start of the study. Random blocks will be stratified by GMFCS level (ambulatory with/without an assistive device or primarily a wheelchair user).

 Following a risk assessment, the weekly 60-minute intervention informed by current guidelines for exercise in cerebral palsy will be delivered by a physiotherapist and a personal trainer experienced in adaptive fitness for individuals with neurological conditions. The exercise component (30 minutes plus 5 minutes of cool-down) and the educational topics to be presented (15-20 minutes) were co-developed by a group with expertise in cerebral palsy, including physiotherapists, personal trainers, and adults with cerebral palsy. The comparison group will receive the same educational information in printed form at the start of the study.

 Feasibility will be evaluated using a mixed-methods approach informed by Bowen’s feasibility framework.  Quantitative data will be gathered to evaluate demand, implementation, practicality, and efficacy.  Qualitative interviews with participants in the intervention group will provide information on acceptability and adaptability.  Sekhon’s theoretical framework of acceptability (including adverse events) will inform qualitative interviews, and Framework Analysis will be used to analyze the interview data.

 Outcome data will be collected at baseline and 8 weeks using self-report measures (multiple PROMIS questionnaires and the Functional Mobility Scale)

 The study has ethical approval and funding. The research project management group includes collaboration among individuals with lived experience of cerebral palsy, physiotherapists, and personal trainers with expertise in cerebral palsy, which is a strength of the protocol.

**Contribution to the field**

 While physical activity and exercise recommendations are similar for adults with and without cerebral palsy (CP), adults with CP face disproportionate barriers to accessing community-based programs that align with evidence-informed guidelines. This protocol addresses a critical implementation gap by evaluating an online exercise-delivery model that will be both adaptable and scalable, potentially offering practical insights that other providers and health systems could leverage.

 The study's co-design approach is a notable strength, engaging key partners and allies, including adults with cerebral palsy, personal trainers, physiotherapists, and CP content experts, throughout the research process from initial design through data analysis, interpretation, and dissemination planning. This participatory framework, combined with the intentional recruitment of participants across all Gross Motor Function Classification System (GMFCS) levels, enhances both the ecological validity and practical relevance of the findings. Such meaningful engagement with end-users and clinical partners positions the study to generate actionable insights that are more likely to translate into real-world implementation.

 The study's strength lies in its potential to simultaneously address multiple access barriers, including transportation, facility accessibility, and program/provider availability, while delivering evidence-informed exercise programming at appropriate intensity levels that can be sustained in participants' home environments. This approach has implications beyond immediate outcomes. It may help reduce secondary health complications associated with physical inactivity in this population, promote health equity by expanding access to tailored programming, and inform more efficient resource allocation within healthcare systems serving adults with CP.

 My additional comments primarily focus on improving the clarity of the background rationale and methods to facilitate replication of the study by other researchers.

**Abstract**

 Consider adding a sentence to the conclusion stating that this feasibility study will inform a larger, fully powered RCT focused on the efficacy of the exercise intervention (and acceptability? Or evidence will be generated?)  This is discussed at the end of the protocol, but it is not clear in the abstract or early in the manuscript.

**Background**

 The need for this study and the research aim is logically described in the background which provides information about the unmet need for community-based exercise for adults with CP, potential for mitigating medical and lifelong progressive impairment related to ageing with CP,  known barriers to accessing exercise programs, and the benefits and opportunities posed by virtual exercise programs. The social aspect of a group exercise program delivered virtually is also an important benefit of the intervention design, and explicitly identifying the link between group exercise and several planned quality of life outcomes in the Background would strengthen the rationale for this study.

 Information on the existing evidence regarding exercise for individuals with CP, including appropriate frequency (dose) and intensity, would add to the rationale for this study and strengthen this section. In addition, emerging evidence suggests that high-intensity or ballistic (power) training may improve upper- and lower-extremity muscle strength and functional mobility (particularly walking) more than traditional progressive resistive strength training or task-specific training, because rapid force generation is necessary for many daily activities.  [Ref 1-3]  Although it is not clear if this type of strength training is included in the planned exercise intervention in this study, this section would be strengthened by a discussion of different exercise recommendations, including aerobic, strength, and flexibility training, for individuals with CP across.  Specifying the focus of the planned training exercises (which are provided only 1x/week) and providing a rationale for that focus would enhance the study's rationale and replicability.

 What does CP-EXCEL stand for? Although it is best practice to name interventions in feasibility and implementation studies, it is unclear whether this acronym refers to the overall study (and eventual fully powered RCT) or the exercise intervention.  Consider defining this to improve clarity in this section.

*Study Aim*

 Although the primary aim of this study is to investigate the feasibility of the exercise intervention, the study appears more complex due to the manner in which the education is delivered.  An exercise-plus-education intervention is compared with a non-equivalent educational approach (printed materials delivered all at once vs. small chunks delivered in an interactive session over 8 weeks). The study is comparing the efficacy of two educational approaches: exercise plus education versus education alone. Consider revising the study aim to clearly identify these different aspects of the study and how they relate to what appears to be a secondary aim of gathering data to inform a larger, fully powered RCT.

**Methods**

*Participants and Recruitment*

 The inclusion of participants across all levels of functional mobility is a strength of this study. However, it is not clear how the inclusion/exclusion criteria will be determined from the screening questions.  How will unstable medical conditions be determined from the screening questionnaire? How will the researchers determine an individual’s inability to access the platform or follow instructions based on intellectual disability based on the screening questionnaire?  If individuals have an intellectual disability, how will the researchers ensure consent to participate? 

*Randomization*

 The description of the concealed allocation procedure is clear and replicable.

*Risk Assessment*

 Consider describing how individuals who require assistance from their support system will engage with the risk assessment.  Also, will participants be withdrawn from the study if their PAR-Q responses or safe environment assessment indicate they are not appropriate for the online exercise intervention? If yes, will this result in additional participants being added to the study after the initial randomization?

 Consider providing more details on the virtual assessment of balance using the 4-stage balance test and the 5 Times Sit-to-Stand test in the home setting, or discussing the lack of evidence for virtual administration of body structure/function level measures [REF 4], the rationale for selecting these measures, and any adaptations needed when administering them virtually. This could include discussing protocols for involving the participant’s support system and planned adaptations to ensure reliability and validity in an online assessment. Also consider elaborating on how this information will be interpreted within the risk assessment (e.g., to modify the exercise program or exclude individuals from the study).

*Online Exercise Program*

 The rationale for a focus on mobility, strength, and balance is sound. The collaboration of a physiotherapist and a personal trainer in delivering the program is a strength.  Consider providing more detailed information on how the listed example exercises will be cued/performed to improve the replicability of this exercise program. For example, for the listed strength exercises, will the emphasis be on speed (power), submaximal muscle force, endurance, or a combination? Or is the overall approach task-specific functional exercises?  Will the exercise instructors encourage 1 repetition max intensity? Or how will the intensity of the exercise be determined?

 Consider clarifying whether balance is explicitly targeted in these exercises or is part of the functional activity/exercises in standing.  How will balance be addressed in seated exercises (in a supportive chair as described in the risk assessment)? If balance is explicitly targeted, please provide examples of these exercises, as the examples provided appear to focus primarily on strength training.

*Replicability of the intervention*

 Documenting the intervention and control interventions using the Template for Intervention Description and Replication (TIDieR) checklist and guide ( https://www.equator-network.org/reporting-guidelines/tidier/ ) would be important for providing a robust description of this exercise intervention. This information would also enhance providers’ or other researchers' ability to determine whether the intervention is appropriate for their clients or study population, particularly for individuals with significant upper-extremity, postural-control, or intellectual impairments.

 Consider providing more information on the rationale for the substantially different educational interventions in the intervention and comparison groups. Alternatively, could the education for the comparison group be delivered in smaller chunks, aligned with the intervention group's content, over 8 weeks? To enhance replicability, it is important to provide a more detailed description of the educational intervention for the control group (e.g., similar topics, but how will it be delivered? How will the researchers ensure that individuals engage with the educational materials, especially if they require support to access or fully understand them?)

 Also, consider elaborating on what physical activity/exercise participants in both groups will be asked to perform between sessions. Will participants in the exercise group be asked to complete additional sessions each week for 8 weeks? Will individuals in the control group be encouraged to engage in similar levels of exercise/physical activity through the educational materials? Current recommendations for physical activity and exercise are the same for all adults, regardless of physical ability (at least 3 days and/or 150 minutes per week of moderate-to-vigorous activity); however, this exercise intervention is delivered once per week. It is unclear how information about additional activity/exercise will be gathered and interpreted in relation to the proposed outcomes of this study.

 Will individuals in the control group have access to the intervention after the feasibility study? How will these individuals, who are interested in a virtual exercise program, benefit from the study? Given the focus on challenges with inequitable access to community exercise programs presented in the Background, it is important to address this issue in the Methods.

*Outcomes*

*Baseline demographic data*

 Consider reporting additional condition-specific participant classifications to enhance the interpretation of subgroup outcomes (e.g., ambulatory versus wheelchair users). Suggestions include the Manual Ability Classification Scale (MACS), Communication Function Classification Scale (CFCS), and Eating and Drinking Classification Scale (EDACS). This is important because it could inform both the adaptations needed during exercise and educational interventions and the interpretation of self-reported mental and social outcomes related to efficacy. What procedures will you use to gather this data if participants are not aware of their these classifications?

 Physical (functional mobility and pain), mental, and social outcomes will be measured using the FMS and PROMIS questionnaires.  These self-report measures have been validated among individuals with CP and are appropriate for assessing the intervention's impact on the range of secondary effects of aging with CP described in the Background. Both the FMS and PROMIS are self-report (or caregiver report) measures.  Will a distinction be made between individuals who can complete these questionnaires independently and those who may do so with support from their support system?

 Consider clarifying whether the baseline assessment is virtual. Including the personal trainer in the risk and baseline assessment is a strength of the study.  The inclusion of individuals with lived experience in the development and piloting of these tools also enhances the study's relevance, rigor, and meaningfulness, which may facilitate future knowledge translation efforts.

*Implementation*

 The sentence “Completion of exercise programme to evaluate fidelity of intervention content by asking participants in the intervention group at each week and control group at the follow-up assessment.” is unclear.  What are participants reporting on here? This question relates to the earlier comment regarding specific recommendations for physical activity or exercise for both groups.  Should they be doing things on their own outside of the exercise class? Is this more than 1x/week?

 Outside of the qualitative interviews, how will you track adaptations made *during *the intervention program? This is an important aspect of implementing (and understanding the feasibility of) an intervention.

*Practicality*

 If the control group also receives exercise or physical activity recommendations in the educational materials, will they also be asked about adverse events?

*Acceptability and Adaptability (Semi-Structured Interviews)*

 Consider including the initial interview guide as an additional file to facilitate replication of the application of the acceptability framework for data collection and analysis. This file would also clarify how adaptability is being assessed using qualitative data.  Will these interviews also include questions specific to the intervention's efficacy components (education vs. exercise)? This aspect is important because the education in the intervention group differs from that in the control group.

 Will the PPI be involved in piloting the interview guide? If so, consider describing this, as it enhances the rigor of the qualitative methods. The inclusion of PPI throughout the study methods and as co-authors on the paper is a strength.

*Efficacy*

 Table 3: FMS is not mentioned in the narrative- consider adding a description and rationale for this tool. PROMIS scales are mentioned in the narrative but not cited in the table. Will FMS be readministered at 8 weeks? Or could this be added to the baseline data collection?

 In addition, it is important to include a more robust description of each measure, its psychometric properties, its relevance for individuals with CP, and the rationale for selecting it for this study, and how you will interpret this data (e.g., what constitutes the minimum clinically important difference?).  There are numerous questionnaires, each with numerous items that participants will self-assess twice during the study.  It is important to describe how you will address the potential for “survey fatigue” and/or the inflation of self-assessment ratings following the intervention over a short period.  

 Will data from both groups be blinded for analysis for the pre-post self-assessments? If yes, consider describing who will complete this task and how identifiable and blinded data will be stored in the protocol, as this enhances the study's rigor and replicability.

*Withdrawals*

 Consider describing what is meant by “standard and usual care” since what care (physiotherapy or other activities) the participants are receiving before the study does not appear to be included in the screening or risk assessment described in this protocol. Standard and usual care also vary geographically within and across regions and countries.

**Discussion**

*Dissemination plan.*

 Consider strengthening this section by including plans to co-develop dissemination strategies (publications, presentations, infographics, or other KT tools) for different target audiences (e.g., individuals with CP and their support systems, personal trainers, policymakers, and other fitness professionals) in collaboration with the PPI group.  In addition, consider planning to identify potential barriers and strategies to support the implementation of the exercise program among both fitness professionals and healthcare providers.

Is the study design appropriate for the research question?

Yes

Is the rationale for, and objectives of, the study clearly described?

Yes

Are sufficient details of the methods provided to allow replication by others?

Partly

Are the datasets clearly presented in a useable and accessible format?

Not applicable

Reviewer Expertise:

Implementation Science Capacity Building, Implementation Practice, Knowledge Brokering, Adults with Childhood Onset- Disability, Inclusive Fitness, Qualitative, Mixed Methods, and Participatory Research in Pediatric Rehabilitation.

I confirm that I have read this submission and believe that I have an appropriate level of expertise to confirm that it is of an acceptable scientific standard, however I have significant reservations, as outlined above.

## References

[1] : Does Home-Based Progressive Resistance or High-Intensity Circuit Training Improve Strength, Function, Activity or Participation in Children With Cerebral Palsy?. *Archives of Physical Medicine and Rehabilitation* .2018;99(12) : 10.1016/j.apmr.2018.06.010 2457-2464.e4 10.1016/j.apmr.2018.06.010 30473019

[2] : Differential Adaptations of Muscle Architecture to High-Velocity Versus Traditional Strength Training in Cerebral Palsy. *Neurorehabilitation and Neural Repair* .2013;27(4) : 10.1177/1545968312469834 325-334 10.1177/1545968312469834 23292847

[3] : Community-Based Upper Extremity Power Training for Youth with Cerebral Palsy: A Pilot Study. *Physical & Occupational Therapy In Pediatrics* .2020;40(1) : 10.1080/01942638.2019.1636924 31-46 10.1080/01942638.2019.1636924 31282292

[4] : Outcomes and standardized tools in telehealth physical therapy for children with cerebral palsy: A scoping review using theICF framework. *Developmental Medicine & Child Neurology* .2025; 10.1111/dmcn.70006 10.1111/dmcn.70006 41164905

# Response

**Published:** 2026-04-20

**Authors:** Manjula Manikandan

**Affiliations:** Royal College of Surgeons in Ireland, Ireland

Authors response

 We have updated the conclusion of the abstract to clarify that this study will inform a future fully powered trial, as follows:

 “This study will provide valuable insights into the feasibility of an online exercise programme tailored for adults with CP and inform the design of a future fully powered randomised controlled trial.”

 We have revised the Background section to more explicitly highlight the link between participation in group exercise, including in a virtual format, and improvements in social participation, mental wellbeing, and overall quality of life, as follows:

 “These group-based formats may also support social participation and mental wellbeing, which are closely linked to quality of life outcomes ^16, 17^.”

 We agree that including information on the evidence base for exercise prescription in people with CP strengthens the rationale for the intervention. We have revised the manuscript (Table 3 and accompanying text) to briefly summarise existing recommendations regarding exercise frequency, intensity, and duration.

 Specifically, current evidence suggests that individuals with CP can safely engage in aerobic and resistance exercise at moderate intensity, with programmes typically delivered 2–4 times per week, progressing as tolerated. Resistance training often involves 2–4 sets of 6–15 repetitions depending on ability.

 We have incorporated this information to better justify the design and dosage of the intervention.

 We agree that acknowledging the range of exercise approaches, including emerging evidence on high-intensity and power-based training, strengthens the rationale for the intervention. We have revised the Background section to briefly outline current exercise recommendations for individuals with CP, including aerobic, strength, and flexibility training, and to acknowledge evidence supporting different training modalities.

 The planned intervention in this study focuses on a combination of mobility, strength, and balance exercises delivered at a moderate intensity, consistent with established exercise guidelines for adults with CP and appropriate for a group-based, online feasibility study. While high-intensity or ballistic training may offer additional benefits, the current programme prioritises safety, accessibility, and adaptability across a wide range of functional abilities (GMFCS I–V), which is particularly important in a feasibility context.

 We have clarified the rationale for the exercise frequency and structure, including the once-weekly supervised session supplemented by independent exercise, and how this aligns with existing recommendations. These additions aim to improve transparency, replicability, and justification of the intervention design.

 CP-EXCEL is the name of the study and refers to “ **Cerebral Palsy – A feasibility randomised controlled trial of an online exercise programme for adults with cerebral palsy**.” It does not refer to the intervention itself, but to the overall feasibility trial evaluating the intervention.

 We have revised the manuscript to clearly define CP-EXCEL at first mention and to distinguish between the study and the intervention components to improve clarity for readers.

 The primary aim of this study is to assess the feasibility of delivering an online group-based exercise and education programme for adults with CP.

 The study compares an exercise-plus-education intervention with an education-only control group. Educational content is provided to both groups, with participants in the intervention group receiving supervised exercise alongside education, while the control group receives educational materials during the study period (weekly) and access to exercise materials following completion of the programme.

 We have revised the study aim to more clearly reflect this comparison while emphasising that the primary focus is on feasibility outcomes and gathering data to inform a larger, fully powered RCT.

 We have clarified that screening will involve both a self-reported questionnaire and follow-up interaction with the research team. Medical stability will initially be assessed through self-report of current health conditions and contraindications to exercise, with any concerns clarified through discussion with the research team where required.

 The ability to access the online platform and follow instructions will not be determined from the questionnaire alone, but will also be assessed during initial interaction with the researcher (e.g., risk assessment call), allowing the research team to evaluate communication, cognitive ability, and ability to engage with the programme. Where appropriate, the role of a family member or caregiver to support participation will be considered.

 For participants with intellectual disability, we will ensure that informed consent procedures are appropriate to the individual’s needs. This will include providing accessible study information and, where required, involving a consultee or caregiver in line with ethical guidelines to support the consent process.

 We are pleased that the description of the concealed allocation procedure is clear and supports replicability.

 We have clarified that participants who require assistance will be supported to engage in the risk assessment with involvement from a family member or caregiver where appropriate. This support may include assistance with communication, technology use, or ensuring a safe exercise environment during both the assessment and intervention.

 We have also clarified that findings from the PAR-Q+ and environmental assessment will be used to determine whether safe participation in the online exercise programme is feasible. Where risks are identified, efforts will be made to mitigate these through adaptations to the environment or exercise programme. However, if safety cannot be ensured despite these adaptations, participants will not proceed to the intervention component of the study.

 As this is a feasibility study, participants who are unable to safely participate will not be replaced following randomisation. This approach allows us to capture important feasibility data regarding eligibility, safety, and implementation, which will inform the design of a future fully powered trial.

 We have expanded this section to provide additional detail on how the 4-stage balance test and 5 Times Sit-to-Stand test will be conducted in a home-based, online setting. Specifically, we have clarified that standardised instructions will be provided, and adaptations (e.g., use of stable supports, positioning of the camera, and involvement of a support person where required) will be implemented to enhance safety and consistency.

 We have also acknowledged that while these measures have demonstrated reliability and have been used in remote contexts, there is limited evidence for their formal validation in adults with CP when administered. This limitation is now explicitly stated.

 Finally, we have clarified how findings from these assessments will be interpreted within the risk assessment process. Specifically, results will be used to inform exercise prescription (e.g., selection, intensity, and level of support required) and to identify any safety concerns. These assessments will not be used in isolation to exclude participants, but rather to guide individualisation of the intervention and ensure safe participation.

 These additions have been incorporated into the Methods section to improve clarity, replicability, and transparency.

 We have clarified that the exercise programme primarily focuses on functional, task-oriented exercises targeting mobility, strength, and balance. The approach emphasises controlled, submaximal effort and movement quality, rather than maximal or explosive (power-based) training. Exercises are designed to be adaptable across a wide range of abilities and are progressed based on individual capacity.

 Exercise intensity will not be prescribed using one-repetition maximum testing. Instead, it will be determined based on individual ability, with participants encouraged to work at a moderate intensity that is challenging yet safe. Progression will be guided by the physiotherapist and personal trainer through adjustment of repetitions (6–15), sets (2–4), range of movement, support level, and exercise complexity. Verbal cueing and demonstration will be used to guide correct technique and ensure safe performance.

 These clarifications have been incorporated into the intervention section to improve transparency and replicability.

 We have clarified that balance is both explicitly targeted and integrated within functional exercises. In standing exercises, balance is challenged through activities such as sit-to-stand, squats, side stepping, toe taps and tasks that involve weight shifting or reduced base of support.

 For participants completing exercises in sitting, balance will be addressed through trunk control and postural stability activities, such as unsupported sitting, reaching tasks, and dynamic upper limb movements that challenge stability within a safe and supported environment (e.g., using a stable chair).

 We have also added examples of balance-focused exercises and clarified how these are adapted across different functional levels. These updates have been incorporated into the intervention section to improve clarity and ensure the role of balance training within the programme is clearly described.

 We have reviewed the intervention and control descriptions in line with the TIDieR checklist and ensured that all relevant items (e.g., rationale, materials, procedures, provider, mode of delivery, dose, and tailoring) are clearly addressed within the manuscript. We have also added a statement to indicate that the intervention is reported in accordance with TIDieR to support transparency, replicability, and external validity.

 The educational content is consistent across both groups and is delivered weekly via email over the 8-week period in the control group, aligning with the topics covered in the intervention group. The key difference lies in the mode of delivery: the intervention group receives education interactively within the supervised sessions, while the control group receives the same content in a self-directed format. This approach was chosen to reflect a pragmatic comparison between an exercise-plus-education programme and an education-only condition, while maintaining feasibility and minimising participant burden.

 We have expanded the description of the control group to clarify the mode and timing of delivery, the accessibility of materials, and the availability of support where needed. As this is a feasibility study, engagement with educational materials in the control group will not be formally monitored to reduce burden; however, this will inform future trial design.

 These clarifications have been incorporated into the Methods section.

 We have clarified that participants in the intervention group will be encouraged to complete the prescribed exercises independently at least twice per week in addition to the weekly online class. To support this, participants will be provided with access to private exercise videos demonstrating the same exercises performed during the supervised sessions. Completion of these additional sessions will be captured through a brief self-reported survey.

 Participants in the control group will be asked to continue their usual activities during the study period and will not be prescribed a structured exercise programme. While educational materials include general information on physical activity, participants will not be specifically instructed to achieve defined exercise targets, in order to maintain the comparison between the exercise-plus-education intervention and an education-only condition. The same exercise videos will be shared with the control group following completion of the 8-week programme.

 As this is a feasibility study, detailed quantification of overall physical activity levels will not be undertaken. However, self-reported adherence to the prescribed exercises in the intervention group will be used to inform interpretation of outcomes and to guide the design of a future fully powered trial.

 These clarifications have been incorporated into the Methods section.

 This has been addressed in the Methods section (Control group), where we specify that participants in the control group will receive access to the exercise materials and be offered the opportunity to participate in an online exercise class following completion of the 8-week programme. This approach ensures that all participants have the opportunity to benefit from the intervention while maintaining the integrity of the study design.

 We have clarified this in the manuscript to improve transparency.

 While we acknowledge that inclusion of measures such as MACS and CFCS could provide additional context regarding upper limb function and communication abilities, these data will not be collected in the current study. As this is a feasibility study, we have prioritised minimising participant burden and focusing on key feasibility outcomes. In addition, all data collected in this study are self-reported, and we considered that reliable classification using MACS- or CFCS-like systems would require clinician assessment or validated structured administration. The intervention is designed to be adaptable to individual needs, and exercise modifications will be guided by participant ability and real-time assessment rather than formal classification.

 We acknowledge that inclusion of condition-specific classifications such as MACS, CFCS, or EDACS could provide additional context for interpreting outcomes and informing adaptations.

 However, these data will not be collected in the current study. As this is a feasibility study, we have prioritised minimising participant burden and focusing on key feasibility outcomes. In addition, all data collected are self-reported, and we considered that reliable classification using systems such as MACS or CFCS would require clinician assessment or validated structured administration, which is beyond the scope of this study.

 The intervention is designed to be adaptable to individual needs, and exercise modifications will be guided by participant ability, functional presentation, and real-time assessment rather than formal classification.

 We have clarified that baseline data collection will be conducted via an online survey (REDCap), and that the risk assessment will be completed virtually via video call.

 We appreciate the positive feedback regarding the involvement of both the physiotherapist and personal trainer in the risk assessment process, as well as the contribution of individuals with lived experience in the development and piloting of study materials. We agree that these elements strengthen the relevance, rigour, and potential for knowledge translation of the study.

 We have revised this section to more clearly describe how completion of the exercise programme will be assessed. Participants in the intervention group will be asked to report, via a brief weekly self-reported survey, whether they completed the prescribed exercises independently at least twice per week in addition to attending the online class. This reflects the intended frequency of exercise participation (i.e., more than once per week).

 These clarifications have been incorporated into the manuscript to improve clarity and alignment with the intervention description.

 In addition to data collected through qualitative interviews, we have clarified that adaptations made during the intervention will be documented prospectively by the researcher delivering the programme. This will include recording any modifications to exercises, delivery format, or participant support made in response to individual needs or emerging challenges during sessions. These records will be reviewed regularly and used to inform ongoing refinement of the programme across the 8-week period.

 This process will complement the qualitative data by providing a structured account of how the intervention is adapted in practice, thereby enhancing transparency, external validity, and knowledge translation.

 While the control group will receive educational materials that include general information on physical activity, they will not be prescribed a structured exercise programme during the study period.

 Adverse events will be assessed in both groups at each assessment point to ensure participant safety and to capture any issues that may arise, regardless of group allocation. However, adverse event monitoring is not linked to prescribed exercise in the control group, but rather reflects overall participant safety during the study period.

 We agree that providing the interview guide will enhance transparency and support replication. The semi-structured interview guide will be made available as a supplementary file alongside the study findings.

 The interview guide has been designed to explore both acceptability and adaptability of the intervention, including participants’ experiences of the exercise and educational components and how these were delivered. Interviews will include questions that capture perceptions of both components and their interaction.

 We would like to clarify that the educational content is consistent across both the intervention and control groups; however, it differs in mode of delivery (interactive versus self-directed).

 Public and Patient Involvement (PPI) contributors will be involved in piloting the semi-structured interview guide. Their input will be used to refine the wording, relevance, and accessibility of the questions to ensure they are appropriate and meaningful for adults with CP.

 We agree that this strengthens the rigour of the qualitative methods, and this has now been clarified in the manuscript.

 The FMS is now described in the narrative alongside the PROMIS measures, including its purpose in assessing mobility across distances and use of assistive devices, and the rationale for its inclusion. We have also ensured that PROMIS measures are consistently referenced in both the narrative and Table 3.

 To clarify timing, the FMS will be collected at both baseline and 8-week follow-up as part of the survey assessments, allowing changes in mobility to be explored over time.

 These updates have been incorporated into the Efficacy section to improve clarity and consistency.

 We have expanded the Efficacy section to provide additional information on the PROMIS measures and the Functional Mobility Scale (FMS), including the domains assessed, scoring approaches, psychometric properties, and their relevance for adults with CP. We have also clarified the rationale for selecting these measures, including their feasibility for remote administration and prior use in neurological populations.

 Regarding interpretation, we have specified that outcomes will be interpreted descriptively in line with the feasibility design, with reference to established PROMIS scoring frameworks and, where available, published guidance on clinically meaningful change. Given the exploratory nature and sample size of this feasibility study, estimation of minimum clinically important differences will be interpreted cautiously and used primarily to inform the design of a future fully powered trial.

 We also acknowledge the potential for survey burden. To mitigate this, we have selected validated PROMIS short forms to minimise the number of items while capturing key domains relevant to the intervention. Data collection is limited to two timepoints (baseline and 8 weeks), and surveys will be administered online to allow flexible completion.

 Due to the nature of the study and the use of self-reported outcome measures collected via REDCap, blinding of group allocation for analysis will not be implemented.

 Baseline and follow-up data will be collected electronically via REDCap and downloaded for pre–post analysis by the researcher. As this is a feasibility study with exploratory analyses, the primary focus is on assessing feasibility parameters rather than minimising bias in effectiveness estimates.

 In this study, “standard and usual care” refers to any healthcare, physiotherapy, exercise, or community-based services that participants would typically access independently as part of their routine care. Participants in both groups will be asked to continue their usual activities and will not be instructed to modify or stop any existing care or physical activity.

 As this is a feasibility study, detailed data on participants’ usual care will not be systematically collected; however, variability in access to services is acknowledged and reflects real-world conditions.

 This has been clarified in the manuscript to improve transparency.

 We have strengthened the Dissemination section to specify that dissemination activities will be developed in collaboration with the PPI group and tailored to different audiences, including adults with CP, families, health professionals, and wider stakeholders. This includes a range of outputs such as peer-reviewed publications, presentations, lay and executive summaries, webinars, and online materials. We have also included plans to provide an accessible summary of findings to participants, co-developed with adults with CP on the research team.

 In addition, we have clarified that intervention materials will be shared to support replication and implementation, and that study findings will inform future work to address barriers and support uptake among relevant stakeholders.

 These updates have been incorporated into the manuscript.

# Reviewer report: Reviewer response for version 1

**DOI:** 10.21956/hrbopenres.15562.r49266

**Published:** 2025-09-03

**Authors:** Margaret Wallen

**Affiliations:** 1 Australian Catholic University, Sydney, Australia

SUMMARY OF STUDY

 The study serves two purposes: to test the feasibility of the intervention and to test the feasibility of the study for scaling up to a larger fully powered RCT.

 Sixty participants will be randomised, (1.1, random permuted blocks, stratified by GMFCS level) with allocation concealment, following baseline assessment, into an intervention and comparison group. Participants, aged 18+, living in Ireland, from across GMFCS levels are eligible to participate.

 Following a risk assessment, the intervention group will participate in an online, group, physical activity programme combined with health education (one topic per week). The physical activity program will be developed based on existing exercise prescription guidelines for people with cerebral palsy and be adapted for completion standing or seated. Intervention will be delivered by a physiotherapist and specialist personal trainer. The program will run for one hour once per week, for 8 weeks.

 The comparison group will receive educational materials at the start of the study.

 Intervention feasibility will be explored with a combination of quantitative metrics and qualitative interviews with participants in the intervention group, using Bowen’s feasibility framework (including adverse events). Sekhon’s theoretical framework of acceptability will be specifically used to guide qualitative interviews. Qualitative analysis will be guided by Framework Analysis.

 Outcomes will be measured at baseline and end of intervention (8 weeks) using the PROMIS and FMS. Analysis will be by generalised estimating equations.

 The study has funding and ethical approval. The research team is well-known for a body of critical work around physical activity participation with people with disability with an excellent track record for completing high quality and impactful research.

 COMMENTS

 This is clearly an important study to prepare for a fully powered study and to offer more definitive evidence of the feasibility of an intervention delivered in reasonably novel scenarios. An online delivery and inclusion of people functioning across all levels of GMFCS, in combination with health education, aims to make an exercise programme more accessible and, the group delivery, more motivating. These are such valuable aspects of the study. Alignment with known exercise guidelines is also a strength. Involvement of people with cerebral palsy on the research team to assist in study design (particularly intervention design and trial), study implementation, data interpretation and co-development of a dissemination plan is highly responsible and will add to the study quality, meaningfulness and capacity for knowledge translation.

 My feedback is mainly suggesting more detailed reporting of the various aspects of the study, currently there is insufficient information to enable replication of the study by others (this is an HRB Open Research reviewer feedback criteria). There appears no word limit for protocols in HRB Open Research and taking advantage of this to demonstrate the scope and rigour of this work could be beneficial. A detailed protocol can also be an advantage if the target journal for publishing the work has a word limit. 

 ABSTRACT - Conclusion.

 Would you consider specifying what information you will generate for instance, evidence to inform the intervention, to inform a larger fully powered RCT.

 BACKGROUND

 This section provides a logical structured background to the study. The need for the study is carefully justified by linking the lifelong nature of CP, issues arising with aging, need for physiotherapy, access to physiotherapy and the opportunity provided by the pandemic of adopting online options for interventions and social interactions.

 Would the authors consider referencing the new proposed updated description of cerebral palsy by Dan and colleagues: [Reference 1]

 If CP-EXCEL is an acronym or has a meaning to be conveyed, it would be interesting to unpack this for the reader (last para in the Background section). Is it the study or the intervention

 Information about the existing evidence for both aspects of the intervention – exercise and/or education – would strengthen your rationale for the study.

 Study aim.

 The aim of the study is clearly written, however, the complexity of the study emerges as the protocol progresses and a final and critical piece is revealed at the end of the discussion. There is an opportunity to be clear about the nature of this study. It is called a feasibility RCT, which tends to imply a small study to explore feasibility of *implementing the study* - recruitment, methods, time involved, outcome measures etc, before rolling it out on a larger scale. This is not specified until the end of the Discussion.

 However, the methods set a reader up to expect an RCT which has an embedded feasibility study exploring feasibility of *the intervention* (as opposed to feasibility of the study).

 There is not a sample size justification in the methods, which is needed anyway, and would have provided a clue to the nature of the study. It is not until the end of the discussion section that the study is declared as underpowered, but will inform a larger study.

 There is an important opportunity to clarify in the early stages of the protocol, if this is my correct assumption, that this is a feasibility study to prepare for a larger well-informed trial AND it has an embedded element which explores the feasibility of this online intervention.

 Please reference the evidence-based framework for feasibility studies mentioned in the aim.

 METHODS

 Study design.

 You may consider adding detail to the design to give the reader a fuller picture of what they will read, for example, RCT where participants will be randomised to an 8-week exercise plus education program with outcomes measured at baseline and end of intervention,  types of intervention, timing of endpoints, allocation concealment.

 Participants

 One of the strengths of this study is including people who are functioning across all GMFCS levels. This also add complexity to implementation of the study. To assist with replicability of the study in the future, it would be beneficial for readers to understand how the researchers will ensure that people with significant disability and with communication and intellectual disability will be able to provide consent and participate in the intervention, and commensurately, how their support network will be asked to/able to assist them.

 Exclusion criteria.

 Other information which would be advantageous for readers to understand to replicate the study is how unstable medical conditions will be ascertained during the screening process, and how individuals will be screened for severe intellectual disability and inability to follow the study.

 Randomisation.

 The description of the randomisation appears very sound and that the process ensures allocation concealment. If this is the case, then it is worthwhile mentioning this, which will assist others in potentially appraising the risk of bias if included in future systematic reviews and clinical guidelines.

 Risk assessment.

 These queries are also related to replicability of the study and also external validity. Consider describing how the balance tests have previously been used online (any evidence for reliability?) or how you will adapt and complete in a standardized manner. 

 Is there any likelihood that the physiotherapist completing the risk assessment will not be comfortable with the risk assessment, for example, if a safe exercise space cannot be guaranteed, or considering PAR-Q findings? Would the person be ineligible for the study?

 Would you consider adding the risk assessment to Figure 1?

 Could you also clarify if the balance assessments may be used to assist in modifying the exercise programme?

 Description of interventions in each arm of the study

 Again, this comment is relevant to replicability and external validity – to enable others to understand the interventions and how they may be relevant to their own population. A strong recommendation would be documenting the intervention and control interventions using the template for intervention description and replication (TIDieR) checklist and guide: https://www.equator-network.org/reporting-guidelines/tidier/

 The would be particularly relevant given that the intervention you are evaluating is highly complex: consists of two parts (exercise and education), and needs modification for several variables - those with mild to severe GMFCS levels, for implementation in a group program, for those with varying upper limb ability, those with associated impairments (intellectual disability, communication, vision and hearing impairment etc), involvement of support people etc.

 It may be that you will choose to provide this information with the publication of the findings – in which case, it would be helpful for readers to understand that the materials will be available with more information about the feasibility of the intervention.

 Intervention group - exercise program and education

 It would be helpful to understand the rationale for the education program in the intervention group, especially as it is not equivalent to that in the control arm?

 Some practical details which would be helpful to provide are: How will participants allocated to the intervention arm of the study be allocated to groups? How many in each group? Is it feasible to have people with diverse GMFCS levels in the same group? How would you tailor exercise for someone in a wheelchair whilst another person is doing squats?

 What procedures will be in place in case of incident during the online group exercise programme?

 Both groups

 Consider clarifying details about what participants will or won’t be asked to do between sessions, and also will you be recording physical activity in both groups. For instance could the provision of the written education material for the control group provoke increased activity?

 Control group.

 It would be worthwhile clarifying details of the control intervention to aid replicability – for instance, is it provided in a written form for people to self-guide . What will participants in this group be advised about other activity, for instance, to continue with all other current activities? Is there existing evidence that it is likely to effect change on the PROMIS?

 Clarify what happens to the control group after the 8 week assessment, for instance, will they be offered the intervention or not?

 PPI

 Inclusion of adults with cerebral palsy is another strength of this study. This section would be strengthened by describing the ways that people with CP have contributed to the research so far and the impacts on the study. Will you consider reporting PPI involvement using a reporting standard such as the GRIPP2-SF http://dx.doi.org/10.1136/bmj.j3453

 Data collection:

 Condition -specific information. Would collection of MACS-like and CFCS-like information be helpful for readers to understand how to generalise your findings and also to understand the kinds of exercise modifications that may have been needed to accommodate upper limb disability?

 Implementation.

 Can you provide more information about “Completion of exercise programme….”.  For the intervention group for instance, is this about whether they completed all exercises and all sets and reps, and the education program? How will this be recorded? For the control group will you ask about whether they read/engaged with the information provided. This would support replicability of the study.

 Practicality: check wording of this sentence: “Practicality will be assessed by recording adverse events by asking participants in both groups at each assessments.”

 Adaptability

 This is an important consideration for an intervention which is delivered online and also that is to be implemented with people who will have diverse motor and other presentations.

 The interviews with participants will obtain pivotal information on adaptability. I am interested also in how you will record the ways that you actually implement adaptations whilst delivering the intervention. This information will be crucial for external validity and knowledge translation.  Aspects will be included in the planned intervention “manual” but it is likely that additional adaptations will be identified once implementation begins.

 Efficacy

 This section would be strengthened and enhance replication by inclusion of information about the PROMIS and FMS (mentioned in table 3) – what domains do they cover, how many questions, how are they rated, what is the magnitude of the scales and the direction of higher/lower function, what scores will be used for analysis? Also appropriate to include would be their suitability/previous use for adults with CP, and online/remote, and psychometric properties including sensitivity to change. Likewise a justification for using these measures would strengthen this section.

 The FMS is only mentioned in Table 3, so potentially it is not included. If so, however, could it be completed by a blinded assessor and add further to the rigour of your methods?

 Overall, the factors which are planned to understand feasibility of *the *intervention are clear but it would add to the rigour of the reporting to also specify which of the metrics will be used to assess feasibility *of the study in preparation for the larger, fully powered study*.

 Data analysis – efficacy

 This section could be strengthened and tightened by specifying that you are analysing the PROMIS (and FMS). You already know the measure and the scores you will be analysing somyou can also specify the nature of the GEE eg linear/logistic. I note the abstract specifies linear mixed models. Finally, specifying how will you interpret the data and identifying what change would constitute an important difference would be a strong inclusion also – adding to the rigour of your reporting.

 Data analysis – qualitative

 What plans do you have regarding collecting (feasibility/interviews) and analysing and integrating data about the two aspects of the intervention: exercise and health education,  for the intervention group?

 You may like to specify the platforms used to support data analysis, e.g., R, NVivo

 Withdrawals:

 Check this sentence for clarity around timing of withdrawal – eg before the intervention starts or finishes?

 “Participants who withdraw before the intervention will continue to receive standard usual care”

 Will participants be replaced if they withdraw before the start of intervention?

 DISCUSSION

 Dissemination plan. This section could be strengthened by including any plans you have for more active knowledge translation beyond the critical but passive dissemination means of publications and presentations

 Communication with participants

 Do you plan to provide a summary of the study findings to participants including how the study impacted practice – to inform how their involvement impacted the field? This would be a strength and I am sure that participants would welcome this. Such communication could be beautifully co-developed with the people with cerebral palsy on the research team.

Is the study design appropriate for the research question?

Yes

Is the rationale for, and objectives of, the study clearly described?

Yes

Are sufficient details of the methods provided to allow replication by others?

Partly

Are the datasets clearly presented in a useable and accessible format?

Not applicable

Reviewer Expertise:

Cerebral palsy

I confirm that I have read this submission and believe that I have an appropriate level of expertise to confirm that it is of an acceptable scientific standard, however I have significant reservations, as outlined above.

## References

[1] : Proposed updated description of cerebral palsy. *Developmental Medicine & Child Neurology* .2025;67(6) : 10.1111/dmcn.16274 700-709 10.1111/dmcn.16274 40213981

# Response

**Published:** 2026-04-20

**Authors:** Manjula Manikandan

**Affiliations:** Royal College of Surgeons in Ireland, Ireland

We thank the reviewer for their positive feedback and for highlighting the strengths of the study. In response to this comment, we have expanded several sections of the protocol to provide additional methodological detail, particularly regarding the recruitment process, intervention delivery, risk assessment, outcome assessments, and data analysis, to improve transparency and support replication.

 We have revised the conclusion of the abstract to clarify that the findings from this feasibility study will inform the refinement of the intervention and the design of a future fully powered randomised controlled trial.

 “This study will provide valuable insights into the feasibility of an online exercise programme tailored for adults with CP and inform the design of a future fully powered randomised controlled trial.”

 We have updated the background section to include the proposed updated description of cerebral palsy by Dan and colleagues and have revised Reference 1 accordingly.

 We have clarified in the Background section that CP-EXCEL refers to the feasibility randomised controlled trial evaluating the online exercise programme.

 “CP-EXCEL refers to the feasibility randomised controlled trial designed to assess the delivery, acceptability and preliminary outcomes of this intervention.”

 We have expanded the Background section to include additional references describing the existing evidence for both exercise and educational interventions for people with cerebral palsy in order to strengthen the rationale for the study.

 We have revised the Background and Aim sections to clearly state that CP-EXCEL is a feasibility randomised controlled trial and that findings will inform the design of a future fully powered trial.

 “Recognising these challenges and opportunities, the CP-EXCEL study aims to evaluate the feasibility of an online exercise programme for adults with CP. CP-EXCEL refers to the feasibility randomised controlled trial designed to assess the delivery, acceptability and preliminary outcomes of this intervention. Findings from this study will inform the design and conduct of a future fully powered randomised controlled trial. If results indicate the programme is feasible, it may provide a sustainable approach to addressing the unmet need for physiotherapy among adults with CP and improve their health and participation.”

 We have revised the Methods section to clarify that this is a feasibility randomised controlled trial focused on assessing the feasibility of study procedures rather than evaluating intervention effectiveness.

 Specifically, we have updated the Study Design, and Data Analysis sections to emphasise the exploratory nature of outcome assessment and the feasibility objectives of the trial.

 “Study design:

 This is a feasibility randomised controlled trial designed to assess the feasibility of study procedures, including recruitment, randomisation, retention, intervention delivery, and outcome assessment, rather than to evaluate intervention effectiveness. The study is not powered to detect statistically significant differences in outcomes, and any analysis of outcomes will be exploratory in nature.

 Data Analysis:

 To determine the fidelity of the programme, descriptive statistics will be used to report the number of sessions attended by participants and those who completed the exercise contents.

 As this is a feasibility study, analyses of outcomes will be exploratory and are not intended to provide definitive evidence of effectiveness.” 

 We have now included a sample size justification in the Methods section, clarifying that the target of 60 participants (30 per group) is appropriate for a feasibility randomised controlled trial and is not based on a formal power calculation. The sample size is informed by the study aim to assess feasibility parameters and by estimated recruitment rates from previous studies involving adults with CP in Ireland.

 This point has been addressed in response to earlier feedback, with revisions made to the Background, Aim, and Methods sections to clearly state that this is a feasibility randomised controlled trial designed to inform a future fully powered trial, and to clarify the evaluation of the feasibility of the online intervention.

 We have now added the reference to the evidence-based framework for feasibility studies in the Aim section.

  We have revised the Study Design section to provide a clearer overview of the trial, including details on group allocation, the 8-week intervention, timing of outcome assessments, and the feasibility focus of the study.

 “Study design

 This is a feasibility randomised controlled trial designed to assess the feasibility of study procedures, including recruitment, randomisation, retention, intervention delivery, and outcome assessment, rather than to evaluate intervention effectiveness. Participants will be randomised to either an 8-week online exercise programme or a control group. Outcome measures will be collected at baseline and immediately post-intervention. The study is not powered to detect statistically significant differences in outcomes, and any analysis of outcomes will be exploratory in nature.”

 We have revised the Participants and Procedures sections to provide additional detail on how individuals with varying levels of disability, including those with communication and intellectual impairments, will be supported to provide informed consent and participate in the study.

 “Participants will be supported to engage in the study using accessible materials and flexible communication approaches (e.g., simplified information or verbal explanation), and may be assisted by a family member or support person where appropriate (e.g., to support consent or participation in online sessions).”

 We have revised the Screening sections to provide additional detail on how exclusion criteria will be assessed. Specifically, we have clarified that unstable medical conditions will be identified through self-report during the screening process and follow-up discussion with the research team where necessary. We have also described how the ability to follow study procedures will be assessed during screening, including consideration of communication and cognitive needs, to determine whether appropriate supports and adaptations can be provided to facilitate participation.

 “All participants will complete a brief screening survey which includes personal details (name, email/phone number), and any health concerns that may prevent them from safely engaging in the exercise programme. Medical stability will be assessed through self-report in this survey, including information on current health conditions and any contraindications to exercise, and clarified through follow-up discussion with the research team where required. The ability to participate in the study will also be assessed during screening and initial interaction with the researcher, considering communication, cognitive ability, and ability to follow instructions. Where appropriate, the potential for support from a family member or caregiver and the feasibility of making adaptations will be considered to facilitate participation.”

 We have revised the Randomisation section to clarify that the allocation sequence is generated and implemented automatically within REDCap, with stratification developed by the study statistician. We have also explicitly stated that allocation concealment is ensured, as the sequence is not accessible to the research team prior to assignment.

 “Randomisation

 Following the baseline assessment, participants will be randomly allocated to either an intervention or control group in a 1:1 ratio. Stratification will be based on their mobility status: GMFCS Level I-III (with or without walking aids) and GMFCS Levels IV-V (who use wheelchairs). The random allocation will be generated by a computer in random permuted blocks of 2 or 4, and implemented automatically within REDCap (Research Electronic Data Capture). Allocation concealment will be ensured as the sequence is embedded within REDCap and not accessible to the research team prior to assignment.”

 We have revised the Risk Assessment section to include references supporting the reliability of the 4-stage balance test and its use in remote assessment. We have also clarified how the assessment will be standardised for use across different GMFCS levels, including the use of consistent instructions and appropriate adaptations to support safe and reliable administration in an online setting.

 “Balance assessment will be conducted using the 4-stage balance test (for GMFCS Levels I-III, as shown in Appendix 2 below), which evaluates static balance ^20^. The 4-stage balance test has demonstrated reliability and has been used in remote assessment contexts ^21, 22^. . While these measures have demonstrated reliability and have been used in remote contexts, there is limited evidence for their formal validation in adults with CP when administered virtually. Although not formally validated for online use in CP, standardised instructions and adaptations will be applied to ensure consistent and safe assessment across GMFCS levels. These assessments will be conducted via video call, with guidance provided on camera positioning to ensure clear observation of movement. Participants will be instructed to perform assessments near a stable support (e.g., chair or wall) to enhance safety.”

 We have clarified in the Risk Assessment section that efforts will be made to adapt the participant’s environment as much as possible to support safe participation. Where potential risks are identified (e.g., through the PAR-Q+ or environmental assessment), these will be addressed through appropriate modifications, guidance, and, where necessary, involvement of a support person. Participants will not be excluded solely based on environmental factors where reasonable adaptations can be made; however, if safety cannot be adequately ensured despite these adaptations, participation in the intervention will not proceed.

 “Where risks are identified, efforts will be made to adapt the environment and exercises to support safe participation. Participants will not be excluded solely based on environmental constraints where reasonable adaptations can be made; however, if safety cannot be ensured, participants will not proceed to the intervention component of the study.”

 We have updated Figure 1 to include the risk assessment step, to provide a clearer overview of the study procedures.

 We have clarified in the manuscript that the results of the balance assessments will be used to inform and adapt the exercise programme as needed, to ensure that it is appropriate and safe for each participant.

 “Findings from the balance and functional assessments will be used to inform the individualisation of the exercise programme, including appropriate exercise selection, level of difficulty, and required adaptations, to ensure safe and appropriate participation.”

 A detailed description of both the intervention and control arms is already provided in the Methods section. We have made minor revisions to further clarify and signpost the content, structure, delivery, and duration of both study arms.

 We have reviewed the intervention and control descriptions in line with the TIDieR checklist and ensured that all relevant items (e.g., rationale, materials, procedures, provider, mode of delivery, dose, and tailoring) are clearly addressed within the manuscript. We have also added a statement to indicate that the intervention is reported in accordance with TIDieR to support transparency, replicability, and external validity.

 We agree that the intervention is complex, involving both exercise and education components and requiring adaptation across a range of participant characteristics. We have revised the manuscript to more clearly describe how the programme will be tailored to accommodate differences in GMFCS levels, upper limb function, and associated impairments, as well as how support persons may be involved where appropriate. These additions aim to improve clarity and support replicability of the intervention.

 “The online exercise class will focus on mobility, strength and balance exercises for major muscle groups, based on the most common reasons that adults with CP seek physiotherapy 12. Participants will complete up to 10-11 exercises in each class. The exercises may be completed in sitting or standing depending on the person’s ability. The intervention will be tailored to individual ability, with exercises adapted based on mobility level (GMFCS I-V), upper limb function, associated impairments (e.g. communication, vision, or hearing) and risk assessment findings, while maintaining a consistent overall structure. Where required, participants may be supported by a family member or caregiver to assist with communication, technology use, or safe participation. Participants will complete between 2 and 4 sets of 6-15 repetitions of each exercise. Examples of exercises include overhead reach, Table or box push-ups, Lateral arm raises, Knee up, hip back or wheelchair dip, side step or hip out, sit to stand, squat, knee straight/bend, heel raises and seated rowing. Exercises will be demonstrated with options and progressions to enable participation within a group setting while accommodating individual differences.”

 We agree that providing detailed intervention materials will support transparency and replicability. We have clarified in the manuscript that these materials will be made available alongside the publication of the study findings, with additional detail informed by the feasibility outcomes in the intervention section and dissemination section.

 We would like to clarify that the educational content is the same for both the intervention and control groups. The difference lies in the mode of delivery: in the intervention group, the educational material is delivered interactively as part of the weekly sessions, whereas in the control group, participants receive the same material at the start of the study without the interactive component. We have clarified this distinction in the manuscript.

 The educational content is consistent across both study groups; however, in the intervention group it is delivered interactively during weekly sessions.

 Control group

 Participants allocated to the control group will receive the same educational materials as those in the intervention group. These will cover topics including exercise, nutrition, mobility changes, fatigue, optimising bone health, pain management, mental health, sleep and physical activity maintenance. These materials will be delivered weekly via email

 Participants will be allocated to the intervention and control arms via REDCap randomisation, with 30 participants in each group. Randomisation is stratified by GMFCS levels (I–III and IV–V) to ensure balanced representation of mobility levels across study arms. Within the intervention arm, participants will take part in group-based sessions, and the programme is designed to accommodate a range of abilities. As described in the manuscript below, exercises will be adapted to suit individual needs, enabling participation of individuals with diverse GMFCS levels within the same group setting.

 We have clarified in the manuscript that procedures are in place to manage any incidents that may occur during the online exercise sessions. These include completion of a pre-participation risk assessment, collection of emergency contact details (next of kin), and ensuring participants have a safe exercise environment prior to participation. During sessions, participants will be supervised by trained professionals, and any adverse events will be recorded and managed in accordance with study procedures.

 Participants in both groups will be asked to continue their usual activities and will not be instructed to modify or stop any existing physical activity. Participants in the intervention group will be encouraged to repeat the exercises independently twice per week in addition to the supervised sessions. We acknowledge that provision of educational materials to the control group may influence behaviour; however, physical activity outside of the intervention will not be formally monitored in this feasibility study. We have clarified these points in the manuscript.

 “Participants will also be encouraged to repeat the exercises independently twice per week between sessions.”

 “Participants in both groups will be asked to continue their usual activities and will not be instructed to modify or stop any existing physical activity.”

 Details of the control condition were already included in the manuscript, including the content, mode (weekly email delivery), and self-directed nature of the materials, as well as guidance for participants to continue their usual activities.

 As this is a feasibility study, no assumptions are made regarding the effect of the control condition on PROMIS outcomes, and any changes will be explored descriptively.

 We have clarified in the manuscript that participants in the control group will be offered the opportunity to take part in an optional online exercise class following completion of the 8-week assessment.

 “Following completion of the 8-week programme, participants in the control group will be offered the opportunity to take part in an optional online exercise class.”

 We have revised the PPI section to more clearly describe how people with CP have contributed to the development of the study and the impact of their involvement on the intervention design and study procedures. We will also consider reporting PPI involvement in line with the GRIPP2-SF reporting guidelines in the final study publication to further enhance transparency and reporting quality.

 While we acknowledge that inclusion of measures such as MACS and CFCS could provide additional context regarding upper limb function and communication abilities, these data will not be collected in the current study. As this is a feasibility study, we have prioritised minimising participant burden and focusing on key feasibility outcomes. In addition, all data collected in this study are self-reported, and we considered that reliable classification using MACS- or CFCS-like systems would require clinician assessment or validated structured administration. The intervention is designed to be adaptable to individual needs, and exercise modifications will be guided by participant ability and real-time assessment rather than formal classification.

 We have clarified in the manuscript how intervention completion will be recorded. For participants in the intervention group, completion will be assessed through attendance at the online group sessions and a brief self-reported survey capturing whether participants completed the exercises independently twice per week. In addition, the number of sets and repetitions performed during supervised group class (6–15 repetitions, 2–4 sets) will be recorded, with progression guided by participant ability over the 8-week programme. Engagement with the educational component will be captured through session attendance.

 For the control group, engagement with the educational materials will not be formally assessed. As this is a feasibility study, we have prioritised minimising participant burden and focusing on key feasibility outcomes related to intervention delivery and adherence. These details have been added to the manuscript to improve clarity and replicability.

 “Adherence to the intervention will be recorded through attendance at online sessions and a brief self-reported survey capturing whether participants completed the prescribed exercises independently twice per week. The number of sets and repetitions performed during supervised sessions will also be recorded, with progression guided by participant ability over the 8-week programme.”

 We have revised the wording of this sentence to improve clarity. It now specifies that adverse events will be recorded by asking participants in both groups at each assessment time point.

 “Practicality will be assessed by recording adverse events reported by participants in both groups at each assessment time point.”

 We agree that adaptability is a key consideration for an online intervention delivered to a population with diverse motor and associated impairments. We have ensured that the manuscript clearly describes how the intervention will be adapted to accommodate individual abilities, including variation in GMFCS levels and other participant needs, to support safe and effective participation.

 We agree that capturing how adaptations are implemented is critical for external validity and knowledge translation. The intervention will be piloted with PPI contributors prior to commencement, and feedback will be used to refine the programme. During the study, qualitative interviews will provide insight into adaptability from the participant perspective. In addition, the researcher delivering the intervention will review session experiences and participant feedback, and implement any necessary adaptations in subsequent group sessions. These adaptations will be documented to inform ongoing refinement of the intervention and future implementation.

 We agree that providing additional information on the outcome measures will strengthen the manuscript and support replicability. We have revised the Efficacy section to include a brief description of the PROMIS and FMS measures below, including the domains assessed, general scoring approach, and interpretation of scores. We have also added a justification for their selection, highlighting their relevance to the intervention, feasibility for remote administration, and suitability for use in adults with CP, along with a brief note on their psychometric properties. Detailed information on specific outcomes and measures remains presented in Table 3 to avoid duplication.

 We would like to clarify that the Functional Mobility Scale (FMS) is included in the study and will be collected as a self-reported measure within the baseline and follow-up surveys. As this is a feasibility study conducted fully online, we have prioritised remote and participant-reported data collection methods to maximise accessibility and minimise burden. For this reason, the FMS will not be administered by a blinded assessor. However, its inclusion as a self-reported measure is considered appropriate for the aims and design of this study.

 We have clarified how each feasibility domain will be assessed by explicitly linking them to predefined metrics, as outlined in Table 1 and how it will inform fully powered trial.

 “Each domain will be assessed using predefined metrics as outlined in Table 1, including recruitment rate (demand), attendance and exercise completion (implementation), adverse events (practicality), qualitative interviews exploring acceptability and adaptability, and changes in physical, mental and social outcomes (potential efficacy). These findings will inform progression to a larger, fully powered trial.”

 We have revised the Data Analysis section to explicitly specify that analyses will be conducted on PROMIS and FMS outcomes.

 We have clarified the statistical approach, specifying that continuous PROMIS outcomes will be analysed using linear mixed models to examine changes over time between groups, while the Functional Mobility Scale (FMS), as an ordinal measure, will be analysed descriptively and explored using appropriate non-parametric or ordinal methods where feasible.

 We have also clarified the approach to missing data. These revisions improve the clarity and reproducibility of the analysis plan.

 The qualitative component of the study is designed to capture participant experiences of the intervention, including both the exercise and health education components. As outlined in the manuscript, semi-structured interviews will explore acceptability and adaptability of the programme, which will encompass both aspects of the intervention for participants in the intervention group.

 Framework analysis will be used to analyse the data, allowing for both exploration of pre-defined domains of acceptability and the identification of emergent themes. We have clarified in the manuscript that the topic guide and analysis will consider participant perspectives on both the exercise and education components, as well as how these interact to influence overall experience and engagement. This approach will support an integrated understanding of the intervention and inform refinement for future implementation.

 We have updated the manuscript to specify the software used for data analysis. Quantitative data will be analysed using Stata (version 18), and qualitative data will be managed and analysed using NVivo 12 Pro (Lumivero, Denver, CO, USA). These details have now been included in the relevant sections of the manuscript to improve clarity and replicability.

 We have revised this section to improve clarity regarding the timing of withdrawal. Specifically, we have clarified that this refers to participants who withdraw prior to commencing the intervention. We have also clarified that participants who withdraw will not be replaced, in line with the feasibility design of the study.

 We have updated the manuscript to highlight active knowledge translation strategies. Findings will be shared with adults with CP, families, and health professionals through publications, presentations, executive and lay summaries, a webinar, and online platforms.

 Yes, we agree that sharing study findings with participants is an important part of knowledge translation and recognising their contribution. We plan to provide a summary of the results in an accessible format, co-developed with adults with CP on the research team, to highlight how participant involvement has contributed to understanding and improving practice. This will be shared with participants after study completion, alongside other dissemination activities such as executive and lay summaries, webinars, and online materials as follows:

 “A summary of the study findings will be provided to all participants in an accessible format. This summary will be co-developed with adults with CP on the research team to ensure it is clear, relevant, and highlights how participant involvement has contributed to understanding and improving practice. This activity complements other dissemination strategies, including executive and lay summaries, webinars, and online materials.”
